# Efficacy and Safety of Treatments for Paroxysmal Nocturnal Hemoglobinuria: A Systematic Literature Review

**DOI:** 10.3390/jcm15114217

**Published:** 2026-05-29

**Authors:** Shreyans Gandhi, Isobel Munro, Victoria Shodimu, Neil Webb, Katharina Pannagl, Anggie Wiyani, Maria-Magdalena Balp

**Affiliations:** 1 Department of Haematological Medicine, King’s College Hospital NHS Foundation Trust, London SE5 9RS, UK; 2 Source Health Economics, London WC1V 7AA, UK; 3 Novartis Pharmaceuticals UK Ltd., London W12 7FQ, UK; 4 Novartis Pharma AG, 4056 Basel, Switzerland

**Keywords:** complement inhibitors, complement 5, complement 3, complement factor B, complement factor D, paroxysmal nocturnal hemoglobinuria, treatment efficacy, safety, systematic review

## Abstract

**Background:** Paroxysmal nocturnal hemoglobinuria (PNH) is a rare blood disorder characterized by complement-mediated hemolytic anemia and thrombosis. The first treatments approved were complement 5 inhibitors (C5is), eculizumab and ravulizumab. Recently approved treatments include pegcetacoplan, iptacopan, danicopan (as an add-on to a C5i), and crovalimab. **Methods:** A systematic literature review (SLR) was conducted to identify clinical evidence on all available treatments. Outcomes evaluated were hemoglobin and lactate dehydrogenase (LDH) levels, transfusion avoidance, Functional Assessment of Chronic Illness Therapy (FACIT)-Fatigue scores, and safety. **Results:** In total, 133 records met the inclusion criteria. Of these, 54 records reporting on 11 Phase 3 trials and 2 extension studies are summarized. Eight trials and one extension study evaluated complement inhibitor (CI)-naïve patients, three trials evaluated CI-experienced patients with residual anemia, and one extension study evaluated both groups. In both patient groups, all treatments led to improved outcomes. **Conclusions:** This SLR is the first to provide an overview of clinical trials assessing the efficacy and safety of all currently approved PNH treatments, which could help inform clinical decisions. Although some head-to-head trials are available, direct comparative evidence remains limited for several comparators, necessitating an indirect treatment comparison (ITC) to assess the efficacy and safety across the treatment landscape.

## 1. Introduction

Paroxysmal nocturnal hemoglobinuria (PNH) is an ultra-rare, clonal hematopoietic stem cell disorder. It is caused by a somatic mutation in the *PIGA* gene, causing deficiency in complement regulatory proteins CD55 and CD59 on the surface of red blood cells (RBCs) [1,2,3,4]. As a result, RBCs become highly susceptible to uncontrolled complement-mediated lysis, with clinical manifestations including hemolysis, anemia, and thrombosis [5]. Prior to the availability of complement inhibitors (CIs), thrombosis was the leading cause of mortality in patients with PNH [6,7,8]. Treatment aims to control hemolysis, increase hemoglobin (Hb) levels, and improve quality of life (QoL) [9].

Six CIs are approved for PNH in the United States and Europe. Approved PNH treatments include CIs targeting component 5 (C5), component 3 (C3), factor B, and factor D of the complement system [10,11,12,13,14,15,16,17,18,19,20,21]. Treatment with eculizumab, an intravenous C5 inhibitor (C5i), administered every 2 weeks, became standard-of-care in many countries following its global approval in 2007 [10,11]. Ravulizumab, another C5i administered intravenously every 8 weeks, was approved in 2018 [12,13]. Treatment with C5i targeting the terminal complement pathway has been associated with reduced intravascular hemolysis (IVH) and thrombotic risk, resulting in improved long-term survival [12,13]. However, up to 82% of patients remain anemic, primarily due to the emergence of extravascular hemolysis (EVH) associated with proximal C3 deposition on RBCs [22,23,24,25,26]. Pegcetacoplan, a subcutaneous C3 inhibitor (C3i) administered twice weekly, was approved by both the Food and Drug Administration (FDA) and European Medicines Agency (EMA) in 2021 as the first PNH treatment targeting the proximal complement pathway [14,15]. In 2023, the twice-daily oral monotherapy iptacopan, which inhibits factor B in the alternative complement pathway, was approved [16,17]. The oral complement factor D inhibitor danicopan, taken three times daily and also targeting the alternative complement pathway, was approved in 2024 as an add-on therapy to eculizumab or ravulizumab [18,19]. Most recently, crovalimab, a novel recycling monoclonal antibody C5i, was approved in 2024 for subcutaneous administration every 4 weeks after an initial intravenous loading dose on Day 1 [20,21].

Despite recent advancements in PNH treatments, guidelines or consensus on incorporating these treatments into clinical practice are lacking. Two systematic literature reviews (SLR) on PNH treatments have been published previously. However, these only included eculizumab, ravulizumab, and pegcetacoplan [27,28]. Furthermore, there was no separate analysis for CI-naïve and CI-experienced patients in da Silva Pires 2023 [28], and safety outcomes were not reported in Lee 2023 [27]. This is the first SLR to identify and report clinical evidence for the efficacy and safety of all FDA and EMA-approved treatments, including recently approved iptacopan, danicopan, and crovalimab, as well as an eculizumab biosimilar, SB12. Clinical evidence was identified from both CI-naïve and CI-experienced adult patients, with a focus on Phase 3 trials. This qualitative SLR provides an overview of the most recent clinical evidence that could help inform clinical decisions, expert consensus, clinical guidelines development, or the design of future quantitative analyses, such as indirect treatment comparisons (ITCs).

## 2. Materials and Methods

An SLR was initially conducted in April 2023 and updated in September 2024, based on a pre-defined protocol, and in accordance with the Cochrane Handbook for Systematic Reviews of Interventions 6.5 [29] and the Preferred Reporting Items for Systematic Reviews and Meta-Analyses (PRISMA) statement [30].

### 2.1. Study Selection Criteria

Assessment for study eligibility was based on protocol-defined population, intervention, comparator(s), outcomes, and study design (PICOS) criteria (Table 1). Studies including patients with PNH who either had not received prior treatment with a CI (CI-naïve) or who were anemic despite CI treatment (CI-experienced) were eligible. In the original SLR, eligible study designs included clinical trials (Phase 2 and above: randomized controlled trials [RCTs], non-RCTs, open-label extensions [OLEs], and long-term follow-up studies), as well as real-world evidence (observational studies, registry studies, and case series). The SLR update only included clinical trials to focus on the pivotal trials of the treatments of interest. Eligible studies were published in English, with no geographical restrictions.

### 2.2. Literature Sources and Searches

Electronic database searches were conducted in MEDLINE, Embase, the Cochrane Database of Systematic Reviews, and the Cochrane Central Register of Controlled Trials. The original SLR included studies from database inception to April 2023, with the update extending to September 2024. Hand-searching was performed on relevant conference proceedings from January 2020 to September 2024, clinical trial registries, and regulatory and health technology assessment agency websites. Bibliographic reference lists of included studies and relevant SLRs and meta-analyses were assessed. Full search details are provided in the Supplementary Materials.

### 2.3. Screening and Extraction

Records were screened against predefined study eligibility criteria by two independent reviewers at title/abstract and full-text screening stages. Disagreements were resolved by a third reviewer. Data from included records were extracted into data extraction tables by one reviewer, with all information checked and validated by a second independent reviewer.

Quality assessment of eligible RCTs was conducted using the Cochrane Risk of Bias 2.0 tool [31], while single-arm trials were assessed using an adapted version of the Critical Appraisal Skills Programme checklist [32].

### 2.4. Outcomes of Interest

Outcomes of interest were Hb levels, transfusion avoidance, lactate dehydrogenase (LDH) levels, breakthrough hemolysis (BTH) events, Functional Assessment of Chronic Illness Therapy (FACIT)-Fatigue scores, and safety outcomes such as serious adverse events (SAEs) as per trial definition and major adverse vascular events (MAVE). Outcomes were extracted and reported using the terminology and definitions from the original publications; detailed definitions are provided in the Supplementary Materials. Key study characteristics are provided in Table 2 and Table 3.

## 3. Results

**Figure 1:**
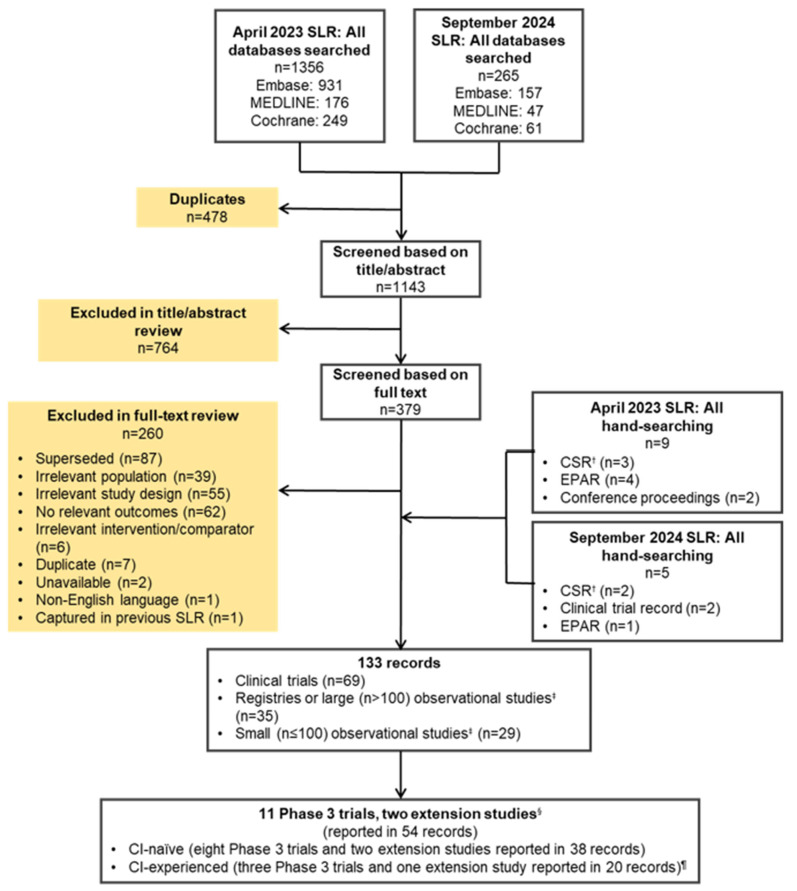
PRISMA flow diagram. ^†^ CSRs were provided by Novartis, the study sponsor. ^‡^ Registry and observational studies were only included in the April 2023 SLR. ^§^ One extension study included CI-naïve patients, and the other extension study included both CI-naïve and CI-experienced patients. ^¶^ Some records reported on both CI-naïve and CI-experienced patient populations. Abbreviations: CI-naïve, complement inhibitor-naïve; CSR, clinical study report; EPAR, European public assessment report; PRISMA, Preferred Reporting Items for Systematic Reviews and Meta-Analyses; SLR, systematic literature review.

A PRISMA diagram illustrating the flow of records through the SLR process is provided in Figure 1. A total of 1621 records were identified through electronic database searches (1356 and 265 from the original SLR and SLR update, respectively). Following screening and hand-searching, 133 records were included in the SLR. Supplementary Table S1 lists all included records. Section 3 of this review focuses on Phase 3 clinical trials, which offer the highest-quality, clinically relevant evidence at the time of the searches. Overall, 54 records reporting on 11 Phase 3 clinical trials and two extension studies were identified and included in this publication (Figure 1). Eight clinical trials (TRIUMPH, SHEPHERD, Study 301, PRINCE, APPOINT-PNH, COMMODORE-2, COMMODORE 3, and Jang 2023 [43] [NCT04058158]) and one extension study (eculizumab extension study) evaluated CI-naïve patients [33,34,36,37,38,40,41,42,43,45]; three clinical trials evaluated CI-experienced patients who remained anemic despite C5i treatment (PEGASUS, APPLY-PNH and ALPHA) [39,40,44]; one extension study included both patient populations (Study 307 OLE) [36].

### 3.1. Risk of Bias Assessment

Eight RCTs [33,37,38,40,41,43,44,45] and three single-arm trials [34,40,42] were assessed for bias, and none were judged to be at high risk (Supplementary Tables S10 and S11). The two extension studies [36,39] were not quality assessed, as they were follow-up analyses of trials that had already undergone quality assessment (Hillmen 2004 [35], SHEPHERD, TRIUMPH, PRINCE, and PEGASUS).

### 3.2. Trials Evaluating CI-Naïve Populations

Eight unique Phase 3 trials and one extension study [33,34,36,37,38,40,41,42,43] (reported in 38 records) were identified in CI-naïve patients, along with one extension study, which included both CI-naïve and CI-experienced patients (Supplementary Table S1) [39]. Five were RCTs [33,37,38,41,43], three were single-arm trials [34,40,42], and two were single-arm extension studies [36,39]. Interventions included eculizumab (TRIUMPH, SHEPHERD, eculizumab extension study) [33,34,36] and eculizumab biosimilar SB12 (NCT04058158) [43], ravulizumab (Study 301) [37], pegcetacoplan (PRINCE, Study 307 OLE) [38,39], iptacopan (APPOINT-PNH) [40], and crovalimab (COMMODORE 2, COMMODORE 3) [41,42]. Among the five RCTs, interventions were compared against eculizumab, placebo, or supportive care [33,37,38,41,43]. Variations in eligibility criteria, efficacy outcomes, and outcome definitions were observed (Supplementary Tables S8–S10).

#### 3.2.1. Efficacy Outcomes

All eight CI-naïve Phase 3 trials reported efficacy outcomes [33,34,36,37,38,40,41,42,43] (Supplementary Table S2).

Five trials had co-primary endpoints: hemolysis control and transfusion avoidance (*n* = 3) [37,41,42]; Hb stabilization and LDH change (*n* = 1) [38]; and Hb stabilization and number of packed RBC (PRBC) units transfused (*n* = 1) [33]. Two trials reported hemolysis [34,43] and one reported hematological response as the primary endpoint [40] (Table 2).

##### 3.2.1.1. Hemoglobin

**Figure 2:**
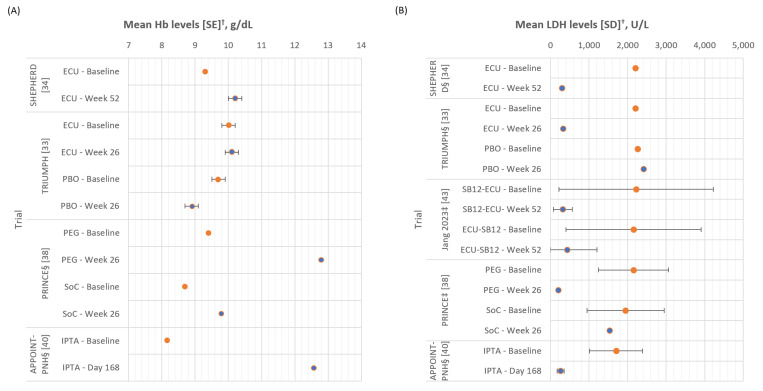

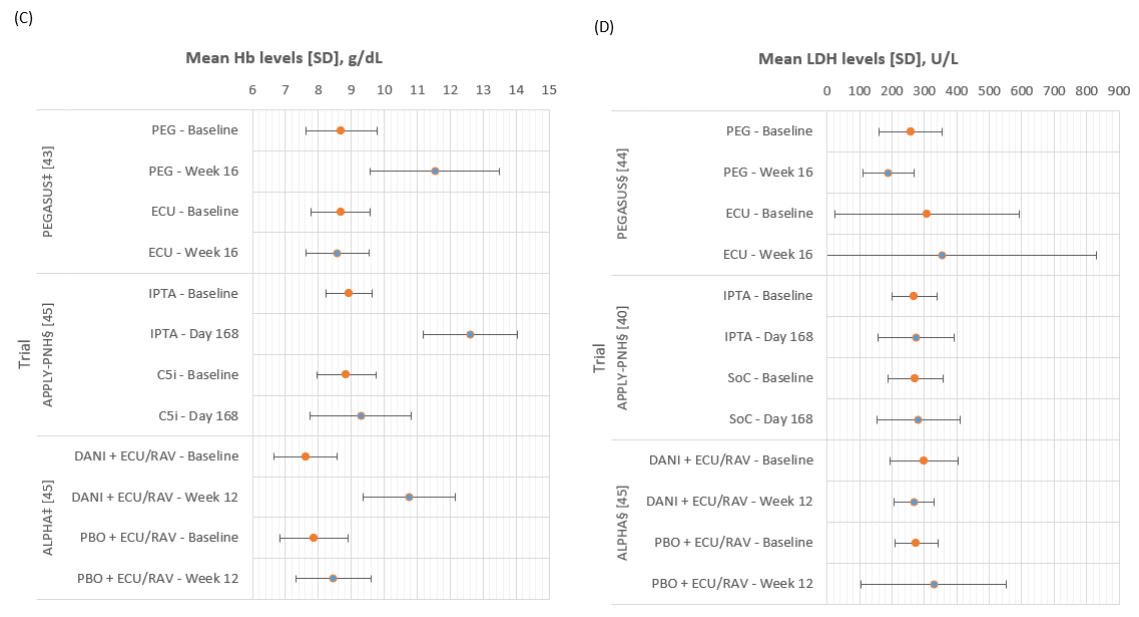
Mean Hb and LDH levels at baseline and follow-up in clinical trials evaluating (**A**,**B**) CI-naïve patients and (**C**,**D**) CI-experienced patients who remained anemic. Note: (**A**) Mean (SE) Hb levels; (**B**) Mean (SD) LDH levels in CI naïve populations; (**C**) Mean (SD) Hb levels; and (**D**) Mean (SD) LDH levels in CI experienced populations. ^†^ SE or SD was not reported for data points without error bars. Statistical measures varied across trials; the most commonly reported measure per outcome was used; ^‡^ Primary endpoint; ^§^ Secondary endpoint. Abbreviations: CI-naïve, complement inhibitor-naïve; DANI, danicopan; ECU, eculizumab; Hb, hemoglobin; IPTA, iptacopan; LDH, lactate dehydrogenase; PBO, placebo; PEG, pegcetacoplan; RAV, ravulizumab; SB12, eculizumab biosimilar; SD, standard deviation; SE, standard error; SoC, standard-of-care.

Figure 2A summarizes baseline and follow-up Hb levels across trials. In TRIUMPH, 49% of eculizumab-treated patients achieved Hb stabilization vs. 0% with placebo at Week 26 (*p* < 0.001) [33]. In Study 301, ravulizumab was statistically non-inferior to eculizumab in terms of Hb stabilization (68.0% vs. 64.5%; *p* < 0.0001) [37]. In PRINCE, pegcetacoplan was superior to supportive care for Hb stabilization (85.7% vs. 0%; *p* < 0.0001) and Hb level change from baseline (CFB) (2.9 g/dL vs. 0.3 g/dL; *p* = 0.0019) [38]. In the single-arm APPOINT-PNH trial, Hb levels were increased vs. baseline in patients receiving iptacopan; with an adjusted mean CFB in Hb level of 4.28 g/dL at Day 168 (95% confidence interval [CI]: 3.87, 4.70) [40]. A sustained increase in Hb levels of ≥2 g/dL from baseline (primary endpoint) and a Hb level of ≥12 g/dL (secondary endpoint) were achieved in 92.2% (95% CI: 82.5, 100) and 62.8% (95% CI: 47.5, 77.5) of patients, respectively [40]. In COMMO-DORE 2, crovalimab was statistically non-inferior to eculizumab in Hb stabilization (63.4% vs. 60.9%; weighted difference: 2.2% [95% CI: −11.4, 16.3]) [41]. Hb stabilization was achieved in 51.0% (95% CI: 36.8, 65.1) of crovalimab-treated patients in the single-arm COMMODORE 3 trial [42].

##### 3.2.1.2. Transfusion Avoidance

In TRIUMPH, the percentage of patients achieving transfusion avoidance at Week 26 was significantly higher with eculizumab vs. placebo (51% vs. 0%; *p* < 0.001) [33]. Jang 2023 (NCT04058158) reported no significant difference in PRBC units transfused between SB12 (eculizumab biosimilar) and eculizumab [43]. In Study 301, ravulizumab resulted in a significantly higher transfusion avoidance than eculizumab at Day 183 (73.6% vs. 66.1%; *p* < 0.0001) [37]. In PRINCE, pegcetacoplan was superior to supportive care for transfusion avoidance through Week 26 (91.4% vs. 5.6%; *p* < 0.0001) [38]. In APPOINT-PNH, 97.6% of iptacopan-treated patients achieved transfusion avoidance between Days 14 and 168 (95% CI: 92.5, 100.0) [40]. Crovalimab was non-inferior to eculizumab in COMMODORE 2 [41], and demonstrated a significant increase in the percentage of patients achieving transfusion avoidance from baseline through Week 25 vs. within 24 weeks of prescreening (51% vs. 0%; *p* < 0.0001) in the single-arm COMMODORE 3 trial [42].

##### 3.2.1.3. Lactate Dehydrogenase

Across trials, all treatments showed reductions in LDH levels at follow-up compared with baseline (Figure 2B). LDH endpoint definitions are provided in Supplementary Tables S8 and S9. In SHEPHERD, mean LDH levels were significantly reduced from baseline with eculizumab at Week 52 (*p* < 0.001) [34]. In Jang 2023 (NCT04058158), least-squares mean (LSM) LDH levels at Week 26 were equivalent between SB12 and eculizumab (284.20 U/L vs. 249.72 U/L) [43]. In Study 301, ravulizumab was non-inferior to eculizumab for LDH normalization (53.6% vs. 49.4%; *p* < 0.0001) and LSM percentage change in LDH levels (difference: −0.83% [95% CI: −5.21, 3.56]; *p* < 0.0001) [37]. In PRINCE, pegcetacoplan was superior to supportive care with respect to LSM CFB in LDH levels at Week 26 (−1870.5 U/L [standard error; SE: 101.0] vs. −400.1 U/L [SE: 313.0]; *p* < 0.0001) [38]. LDH normalization was significantly higher in pegcetacoplan-treated patients compared with supportive care (65.7% vs. 0%; *p* < 0.0001). In APPOINT-PNH, iptacopan reduced LDH levels from baseline at Day 168 (−83.57 U/L [95% CI: −84.99, −82.03]); with 95% of patients achieving LDH normalization [40]. In COMMODORE 2, crovalimab was non-inferior to eculizumab for hemolysis control (79.3% vs. 79.0%; odds ratio [OR]: 1.0 [95% CI: 0.6, 1.8]) [41] and in the single-arm trial COMMODORE 3, crovalimab met its primary endpoint (mean proportion of patients achieving hemolysis control was 78.7% [95% CI: 67.8, 86.6]) [42].

##### 3.2.1.4. Breakthrough Hemolysis Events

The definition of breakthrough hemolysis (BTH) differed across trials; detailed study-specific definitions are provided in Supplementary Table S10. BTH was an efficacy outcome in six trials [34,37,40,41,42,43] (Supplementary Table S4). The proportion of patients with BTH events was 8.2% with eculizumab in SHEPHERD [34], 10.7% vs. 4.0% with eculizumab vs. ravulizumab in Study 301 [37], and 17% vs. 2% with SB12 (eculizumab biosimilar) vs. eculizumab in Jang 2023 (NCT04058158) [43]. No BTH events occurred with iptacopan in APPOINT-PNH [40]. In COMMODORE 2, 14.5% of patients experienced BTH with eculizumab and 10.4% with crovalimab [41], whereas in COMMODORE 3, 3.9% of patients receiving crovalimab experienced BTH [42].

##### 3.2.1.5. FACIT-Fatigue

FACIT-Fatigue is a 13-item patient-reported outcome measure with scores ranging from 0 to 52, where lower scores indicate greater fatigue. FACIT-Fatigue was reported in seven of the eight CI-naïve Phase 3 trials, with the exception of Jang 2023 (NCT04058158) [33,34,36,37,38,40,41,42,43] (Figure 2A and Supplementary Table S3). All treatment arms demonstrated increases in mean FACIT-Fatigue scores, reflecting improvement in patient-reported fatigue (Figure 2A). No formal hypothesis testing was conducted between trial arms.

#### 3.2.2. Safety Outcomes

**Figure 3:**
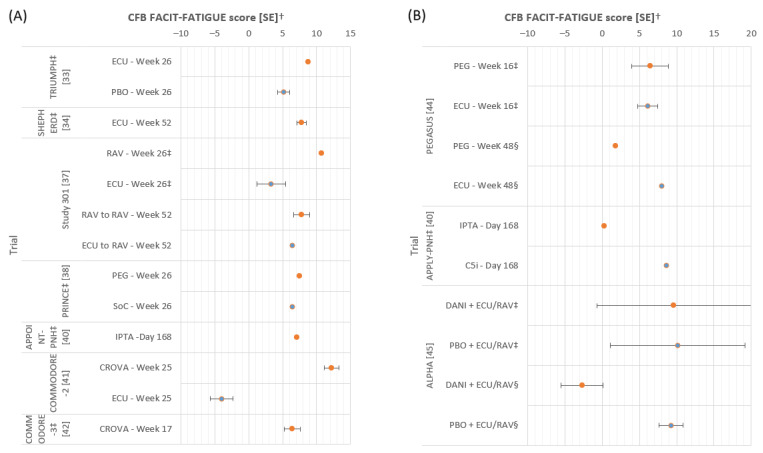

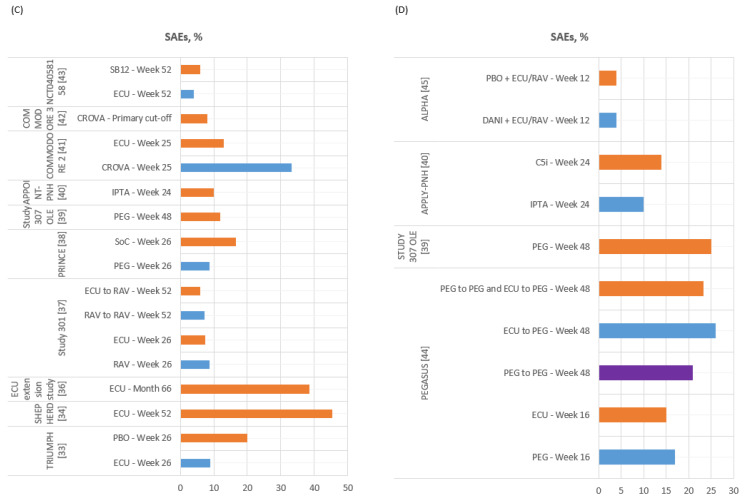
(**A**,**B**) CFB in FACIT-fatigue and (**C**,**D**) SAEs reported at follow-up in clinical trials evaluating CI-naïve and CI-experienced patients. Note: (**A**,**C**) CI naïve populations; (**B**,**D**) CI experienced populations. ^†^ HRQoL outcomes have been sourced from all referenced records per clinical trial in this table. If different timepoints are reported in separate records, it has been indicated which record reports each timepoint; SE or SD was not reported for data points without error bars. Statistical measures varied across trials; the most commonly reported measure per outcome was used; ^‡^ Secondary endpoint; ^§^ SD. Abbreviations: CFB, change from baseline; CI-naïve, complement inhibitor-naïve; CROVA, crovalimab; DANI, danicopan; ECU, eculizumab; IPTA, iptacopan; PBO, placebo; PEG, pegcetacoplan; RAV, ravulizumab; SAE, serious adverse event; SB12, eculizumab biosimilar; SD, standard deviation; SE, standard error; SoC, standard of care.

SAEs were reported in all eight Phase 3 trials [33,34,36,37,38,40,41,42,43] (Figure 3C and Supplementary Table S4). MAVEs were assessed across all trials [33,34,36,37,38,40,41,42,43], with no events reported for pegcetacoplan [38], iptacopan [40], and SB12 [43]. Patients experiencing MAVEs ranged from 0% [33] to 2.1% [34] with eculizumab, 0% [42] to 0.74% [41] with crovalimab, and 1.6% with ravulizumab [37] (Supplementary Table S4).

#### 3.2.3. Extension Studies

Across the eculizumab extension study [36] and Study 307 OLE [39], long-term treatment with eculizumab and pegcetacoplan led to sustained improvements in Hb and LDH levels. The long-term safety results support the safety profiles previously established for both therapies (Supplementary Tables S2 and S4).

### 3.3. Trials Evaluating CI-Experienced Populations

Three Phase 3 RCTs [40,44,45] and an extension study (Study 307 OLE) [39] (reported in 20 records) evaluated interventions for CI-experienced patients who remained anemic despite C5i treatment (Supplementary Table S1). Interventions evaluated included pegcetacoplan vs. eculizumab (PEGASUS, Study 307 OLE) [39,44], iptacopan vs. C5i (APPLY-PNH) [40], and danicopan + C5i vs. placebo + C5i (ALPHA) [45] (Table 3).

#### 3.3.1. Efficacy Outcomes

Efficacy outcomes were reported in all three Phase 3 trials evaluating CI-experienced patients who remained anemic [40,44,45] (Supplementary Table S5). Primary endpoints included CFB in Hb levels [44,45] and hematological response [40] (Table 3).

##### 3.3.1.1. Hemoglobin

Figure 2C summarizes Hb levels at baseline and follow-up across these trials. In PEGASUS, pegcetacoplan showed superior Hb improvement vs. eculizumab at Week 16 (adjusted LSM CFB difference: 3.84 g/dL; *p* < 0.001) [44]. In APPLY-PNH, absolute mean CFB in Hb with iptacopan was superior to C5i at Week 24 (adjusted mean difference: 3.7 g/dL [95% CI: 3.2, 4.1]; *p* < 0.001) [40]. In ALPHA, danicopan + C5i significantly increased Hb vs. placebo + C5i at Week 12 (LSM difference: 2.44 g/dL; *p* < 0.0001) [45].

Hematological response was reported in APPLY-PNH with two definitions: proportion of patients with an increase in Hb levels of ≥2 g/dL from baseline; and proportion of patients with a Hb level of ≥12 g/dL, both in the absence of RBC transfusion. Iptacopan was superior to C5i for both definitions of hematological response (treatment differences in marginal proportions at Day 168, ≥2 g/dL increase: 82% vs. 2%; *p* < 0.001 and ≥12 g/dL increase: 69% vs. 2%; *p* < 0.001) [40]. In ALPHA, when compared with placebo + C5i, danicopan + C5i statistically significantly improved hematological response (≥2 g/dL increase) at Week 12 (60% vs. 0%; adjusted difference: 47% [95% CI: 29, 65]; *p* < 0.0001) [45].

##### 3.3.1.2. Transfusion Avoidance

In PEGASUS, transfusion avoidance was significantly higher with pegcetacoplan than eculizumab (85% vs. 15%; *p* < 0.001) at Week 16 [44]. In APPLY-PNH, iptacopan had significantly higher transfusion avoidance vs. C5i, with a treatment difference of 68.9% (95% CI: 51.4; 83.9; *p* < 0.001) between Days 14 and 168 [40]. In ALPHA, danicopan + C5i demonstrated a significantly higher rate of transfusion avoidance vs. placebo + C5i (83% vs. 38%; adjusted difference: 42% [95% CI: 23, 61]; *p* = 0.0004) at Week 12 [45].

##### 3.3.1.3. Lactate Dehydrogenase

Figure 2D summarizes LDH levels at baseline and at the end of the randomized treatment period. The LDH levels remained below 1.5 × ULN in APPLY-PNH and were sustained from baseline in PEGASUS and ALPHA after switching from C5i. In PEGASUS, pegcetacoplan did not demonstrate non-inferiority to eculizumab for mean CFB in LDH levels at Week 16 (−15 [standard deviation; SD: 43] vs. −10 [SD: 71] U/L); however, more patients achieved LDH normalization (71% vs. 15%) [44]. In APPLY-PNH, iptacopan was not superior to C5i for percentage reduction from baseline in LDH at Week 24 (−3.5% [95% CI: −10.0, 3.4] vs. −2.4% [95% CI: −10.8, 6.7] [40]. In ALPHA, danicopan + C5i demonstrated greater reduction in LDH from baseline vs. placebo + C5i at Week 12 (−23.49 U/L [95% CI: −40.08, −6.90] vs. −2.92 U/L [95% CI: −26.78, 20.93]) [45].

##### 3.3.1.4. Breakthrough Hemolysis Event

BTH was not assessed in ALPHA [45] but was reported as a secondary endpoint in APPLY-PNH [40] and as a safety outcome in PEGASUS [44]. In PEGASUS, BTH occurred in 10% of patients receiving pegcetacoplan vs. 23% receiving eculizumab [44]; in APPLY-PNH, 3.2% of iptacopan-treated patients vs. 17.1% of C5i-treated patients experienced BTH [40].

##### 3.3.1.5. FACIT-Fatigue

FACIT-Fatigue was reported in all three trials evaluating CI-experienced patients [40,44,45] (Figure 3B and Supplementary Table S6). All treatment arms demonstrated increases in mean FACIT-Fatigue scores, reflecting improvement in patient-reported fatigue (Figure 3B).

#### 3.3.2. Safety Outcomes

All three trials evaluating CI-experienced patients reported SAEs [40,44,45] (Figure 3D and Supplementary Table S7). MAVEs were not reported in ALPHA [45]. In APPLY-PNH, one (1.6%) iptacopan-treated patient experienced MAVEs vs. 0% with C5i [40]. In PEGASUS, no patients experienced a MAVE with either pegcetacoplan or eculizumab through Week 16; by Week 48, including the period following the switch from eculizumab to pegcetacoplan in the comparator arm, one patient in each arm (3%) experienced a MAVE [44].

#### 3.3.3. Extension Studies

Consistent with the CI-naïve population, long-term treatment with pegcetacoplan in Study 307 OLE [39] resulted in sustained improvements in hemoglobin and LDH levels among CI-experienced patients. The long-term safety data continued to support the favorable safety profile established in the primary trial (Supplementary Tables S5 and S7).

## 4. Discussion

This SLR summarizes clinical trial evidence for all FDA- and EMA-approved PNH treatments at present, including recently approved iptacopan, danicopan (as an add-on to C5i; ravulizumab/eculizumab), crovalimab, and an eculizumab biosimilar, SB12. It includes pivotal Phase 3 trials assessing efficacy and safety in both CI-naïve and CI-experienced populations.

The SLR was conducted using predefined eligibility criteria and adhered to PRISMA guidelines [30], supporting the robustness of its conclusions. The literature search was conducted according to a predefined protocol and updated in September 2024, with no date restriction applied to full-text published studies. However, methodological limitations remain, such as the exclusion of conference abstracts published before 2020 and non-English language records. The review intentionally focuses on Phase 3 trials, as these represent the highest quality and most clinically relevant evidence at the time of the searches.

In patients with CI-naïve PNH, five distinct Phase 3 RCTs [33,37,38,41,43] were identified, each evaluating a different intervention; eculizumab, ravulizumab, pegcetacoplan, crovalimab, and SB12. Three single-arm trials [34,40,42] evaluated eculizumab, iptacopan, or crovalimab. Two extension studies were also identified: the long-term eculizumab extension study [36] and Study 307 OLE evaluating long-term pegcetacoplan use [39]. Hemolysis control was the most common primary endpoint. All treatments were effective in reducing LDH levels and transfusion dependence. FACIT-Fatigue scores improved across all Phase 3 trials, with a clinically meaningful mean increase of ≥5 points in the intervention arms, indicating improvement in patient-reported fatigue [46].

In CI-experienced patients who remained anemic during C5i treatment, three Phase 3 trials [40,44,45] and one extension study (Study 307 OLE) [39] were identified. Across these trials, follow-up duration and the timing of endpoint assessments varied, which should be considered when evaluating the feasibility and design of both future ITCs and clinical trials. These trials evaluated different interventions, including iptacopan, pegcetacoplan, and danicopan (as an add-on to C5i). The most frequently assessed primary endpoint was CFB in Hb levels, with all treatments leading to increased Hb. LDH levels were well-controlled at baseline in all three trials, reflecting effective IVH management from prior C5i therapy. After switching from a C5i to either pegcetacoplan or iptacopan, LDH levels remained low, indicating sustained hemolysis control. A clinically meaningful increase in FACIT-Fatigue scores was observed across all interventions, resulting in improved QoL. BTH occurred less frequently with iptacopan than C5i (ravulizumab/eculizumab) [40], and with pegcetacoplan than eculizumab [44]. These findings are based on data from pivotal Phase 3 trials, and longer-term follow-up data are only available from the eculizumab extension study [36] and Study 307 OLE evaluating long-term pegcetacoplan use [39].

Compared with previously published SLRs, this SLR is more comprehensive, covering a wider range of treatment options, all approved treatments with published evidence available at the time of the SLR and distinguishing between CI-naïve and CI-experienced populations with residual anemia [27,28]. It is the first to identify evidence for the efficacy and safety of all FDA- and EMA-approved treatments, including eculizumab, ravulizumab, and pegcetacoplan, as well as the recently approved iptacopan, danicopan (as an add-on to C5i), crovalimab, and the eculizumab biosimilar, SB12. Robust clinical evidence was identified in this SLR, demonstrating the effectiveness of these treatments in improving anemia (through increasing Hb levels) and controlling LDH levels. Although treatment with eculizumab, the first approved CI, has resulted in IVH control as well as reduction in thrombosis risk, studies have also shown that treatment with C5i can be associated with the emergence of EVH, which could lead to residual anemia [9,47]. In this context, trials of proximal CIs (pegcetacoplan, danicopan, iptacopan) have demonstrated efficacy in improving Hb levels in affected patients.

This review identified differences in the patient eligibility criteria of included clinical trials, reflecting the varying times at which they were conducted, ranging from 2006 to 2024, and the shifting therapeutic landscape. Key differences include clone size thresholds, LDH-based hemolysis criteria, Hb levels, prior CI exposure, and recent transfusion or symptom history. Moreover, the crovalimab COMMODORE trials recruited adolescent and adult patients [41,42] whereas all other trials only recruited adult patients. These trials were included in the review as only a small number of patients were under 18 years: 2 patients (2.9%) in COMMODORE 2 (both 17 years old), and 3 patients (6%) in COMMODORE 3 [41,42]. An additional trial (COMMODORE 1) was excluded as it did not state whether patients were anemic after prior C5i treatment [48].

Across trials, substantial heterogeneity was observed in efficacy outcome definitions, particularly for hematological response and breakthrough hemolysis, with additional variation in the time points at which these outcomes were assessed (Supplementary Table S10). Of note, the severity of BTH may vary depending on the complement pathway targeted by treatments, the mechanism of inhibition, mode of administration and individual patient characteristics [49]. Nonetheless, this qualitative review has highlighted differences in patient inclusion and exclusion criteria, as well as outcome definitions and assessment time points, which may limit direct comparability across trials. While ITC methods could adjust cross-trial differences, these considerations should inform the feasibility assessment of future ITCs. Additionally, future clinical trials could align outcome definitions and assessment time points at the design stage to improve consistency across studies and facilitate comparative evidence generation.

One of the main limitations in the trials of PNH treatments is the scarcity of large-scale trials due to PNH being an ultra-rare disease; the sample size was <100 patients in most included trials. The number of trials that focus on CI-experienced patients with residual anemia was small; the majority of trials identified were in the CI-naïve population (Table 2 and Table 3). Furthermore, most CI-naïve trials compared treatments either with placebo/supportive care or were single-arm trials. In the CI-experienced population, no head-to-head trials have been conducted between proximal CIs. This likely reflects the parallel timing of their clinical development, whereby other proximal CIs were not yet available when the Phase 3 RCTs were designed. As a result, trials evaluating CIs targeting the proximal complement pathway used C5Is targeting the terminal complement pathway (ravulizumab or eculizumab) as the most appropriate available comparators.

The FDA and EMA approvals of new CIs have expanded treatment options for patients with PNH, offering diverse therapeutic strategies across the United States and Europe, and facilitating broader global access. However, access to these treatments varies between countries, which limits treatment options in clinical practice and influences clinical decision-making. Non-conformity in baseline characteristics and outcome measures across trials, as well as variation in treatment access, may have contributed to a lack of consensus on a unified approach of integrating these treatments into clinical practice. This publication aims to support clinicians by reporting data from globally available RCTs through an SLR, providing a clear and comprehensive overview of clinical efficacy and safety outcomes of approved treatments, to inform evidence-based decision-making. However, in view of the qualitative nature and limitations of this SLR, an ITC or a direct head-to-head RCT is required to quantitatively evaluate the comparative effectiveness and safety between PNH treatments.

## 5. Conclusions

PNH is an ultra-rare disease with an evolving treatment landscape. This SLR is the first to summarize the efficacy and safety of all currently available FDA- and EMA- approved PNH treatments, including eculizumab, ravulizumab, and pegcetacoplan, as well as the recently approved iptacopan, danicopan (as an add-on to C5i), crovalimab, and the eculizumab biosimilar SB12. This SLR provides an overview of trial evidence for these treatments, which could help inform clinical decisions, expert consensus, clinical guidelines development, or the design of future quantitative analyses, such as ITCs. Although some head-to-head trials are available, direct comparative evidence remains limited for several comparators, necessitating an ITC to assess the efficacy and safety across the treatment landscape.

## Figures and Tables

**Table 1:** Eligibility criteria (PICOS).

Characteristics	Inclusion Criteria—Original SLR	Inclusion Criteria—Update SLR	Exclusion Criteria—Update SLR
Population	Adults (aged ≥ 18 years) with paroxysmal nocturnal hemoglobinuria: ◦ who are anemic after treatment with any complement inhibitor ◦ who have not received prior treatment with any complement inhibitor	Adults (aged ≥ 18 years) with paroxysmal nocturnal hemoglobinuria: ◦ ***who have hemolytic anemia*** ◦ who are anemic after treatment with any complement inhibitor ◦ who have not received prior treatment with any complement inhibitor	Pediatric patients (aged < 18 years) where they account for 100% of the study population Mixed populations: If the study includes >20% pediatric patients, the study will be excluded unless outcomes are reported separately for adult patients
Intervention/comparators	Iptacopan (LNP023) Pegcetacoplan (Empaveli, Aspaveli) Eculizumab (Soliris, including biosimilars) Ravulizumab (Ultomiris) Danicopan (ALXN2040, ACH-0144471) Crovalimab (RG6107, SKY59) ***Vemircopan (ALXN2050)*** ^†^ ***Pozelimab (REGN3918)*** ^†^ ***Cemdisiran (ALN-CC5)*** ^†^ NM8074 ARO-C3 KP104 CAN-106	Iptacopan (***Fabhalta***, LNP023) Pegcetacoplan (Empaveli, Aspaveli) Eculizumab (Soliris, including biosimilars) Ravulizumab (Ultomiris) Danicopan (ALXN2040, ACH-0144471) Crovalimab (RG6107, SKY59) ***Ruxoprubart*** (NM8074) ARO-C3 KP104 ***Omoprubart*** (CAN-106)	Interventions/comparators not listed
Outcomes	Efficacy Hb levels, including but not limited to: ◦ Hemoglobin levels at follow-up ◦ Change in hemoglobin levels from baseline ◦ Proportion of patients achieving a sustained increase in hemoglobin levels from baseline Intravascular hemolysis, largely measured by LDH levels, including but not limited to: ◦ LDH levels at follow-up ◦ Change in LDH levels from baseline ◦ Proportion of patients achieving LDH normalization Extravascular hemolysis (largely measured by reticulocytes, including but not limited to: ◦ Reticulocyte count at follow-up ◦ Change in reticulocyte count from baseline Proportion of patients achieving breakthrough hemolysis Transfusion avoidance Transfusion frequency and number of units of PRBCs transfused Spontaneous remission Mortality Safety Major adverse vascular events (inc. thrombosis) Treatment discontinuation Adverse events HRQoL FACIT-Fatigue EORTC QLQ-C30	Efficacy Hb levels, including but not limited to: ◦ Hemoglobin levels at follow-up ◦ Change in hemoglobin levels from baseline ◦ Proportion of patients achieving a sustained increase in hemoglobin levels from baseline Intravascular hemolysis, largely measured by LDH levels, including but not limited to: ◦ LDH levels at follow-up ◦ Change in LDH levels from baseline ◦ Proportion of patients achieving LDH normalization Extravascular hemolysis (largely measured by reticulocytes, including but not limited to: ◦ Reticulocyte count at follow-up ◦ Change in reticulocyte count from baseline Proportion of patients achieving breakthrough hemolysis Transfusion avoidance Transfusion frequency and number of units of PRBCs transfused Spontaneous remission Mortality Safety Major adverse vascular events (inc. thrombosis) Treatment discontinuation Adverse events HRQoL FACIT-Fatigue EORTC QLQ-C30	Outcomes not listed
Study design	RCTs (Phase II and above) Multi-arm non-randomized trials Single-arm clinical trials Open-label extensions or long-term follow-up trials ***Real-world evidence*** ◦ ***Retrospective or prospective observational studies, including cohort studies*** ◦ ***Medical record review/chart review studies*** ◦ ***Claims database analyses*** ◦ ***Patient registry analyses*** ◦ ***Case series***	RCTs (Phase II and above) Multi-arm non-randomized trials Single-arm clinical trials Open-label extensions or long-term follow-up trials	***Real-world evidence*** ◦ ***Retrospective or prospective observational studies, including cohort studies*** ◦ ***Medical record review/chart review studies*** ◦ ***Claims database analyses*** ◦ ***Patient registry analyses*** ◦ ***Case series*** Reviews/editorials/commentaries/letters SLRs/(N)MAs ^‡^ In vitro/animal studies/pre-clinical studies Studies where *N* < 10
Date limits	No restriction	***April 2023–present***	***Pre–April 2023***
Countries	No restriction	No restriction	–
Languages	English language records	English language records	Non-English language records

Note: ***Bold and italics*** indicate changes in inclusion and exclusion criteria between the original and updated SLRs. ^†^ Pozelimab, vemircopan, and cemdisiran were removed as comparators for the SLR update due to their clinical trial programs being terminated. The brand name for iptacopan, and the generic names for both NM8074 and CAN-106 were added for the SLR update. ^‡^ Relevant SLRs/NMAs were included at title/abstract screening stage so their bibliographic reference lists could be hand-searched for relevant studies. Abbreviations: EORTC QLQ-C30, European Organization for Research and Treatment of Cancer Quality of Life of Cancer Patients; FACIT-Fatigue, Functional Assessment of Chronic Illness Therapy-Fatigue; Hb, hemoglobin; LDH, lactate dehydrogenase; (N)MA, (network) meta-analysis; PICOS, population, intervention, comparator, outcomes, and study design; PRBC, packed red blood cell; RCT, randomized controlled trial; SLR, systematic literature review.

**Table 2:** Key study characteristics of clinical trials evaluating patients with CI-naïve PNH.

Clinical Trial Name or Acronym Primary Publication	Study Design; Masking Status; Country	Key Study Inclusion Criteria	Intervention vs. Comparator	Endpoints	Key Analysis Timepoints
Eculizumab clinical trials					
**TRIUMPH** Hillmen 2006 [33]	RCT Double-blind International multicentre	Hb ≤ 10.5 g/dL LDH ≥ 1.5 × ULN Platelet count ≥ 100,000/m^3^ PNH type III erythrocyte proportion ≥ 10% ≥4 transfusions in previous 12 months	ECU (*n* = 43) vs. PBO (*n* = 44)	**Primary:** Stabilization of Hb levels and the number of units of PRBC transfused during that period **Secondary:** Transfusion independence: hemolysis; CFB FACIT-Fatigue (13-item)	Week 26
**SHEPHERD** Brodsky 2008 [34]	Single-arm CT Open-label USA	LDH ≥ 1.5 × ULN Platelet count ≥ 30,000/m^3^ GPI-deficient red blood cell clone (type III cells) ≥ 10% ≥4 transfusions in previous 2 years	ECU (*n* = 97)	**Primary:** Hemolysis; safety (AEs, clinical laboratories, ECG data, and vital signs) **Secondary:** FACIT-Fatigue (13-item); CFB LDH	Week 52
**Eculizumab extension study** (including patients from Hillmen 2004 [35], SHEPHERD, and TRIUMPH) Hillmen 2007 [36]	International multicentre Single-arm extension study Open-label International multicentre	Patients who fully completed Hillmen 2004 [35], SHEPHERD, or TRIUMPH (patients were naïve to treatment when entering the initial pivotal trial before the extension phase)	ECU (*n* = 195)	**Primary:** Thromboembolism (MAVEs) **Secondary:** NR	Week 102
Ravulizumab clinical trials					
**Study 301** Lee 2019 [37]	RCT Open-label International multicentre	Platelet count ≥ 30 × 10^9^/L Red and white blood cells with granulocyte or monocyte clone size ≥ 5%	RAV (*n* = 125) vs. ECU (*n* = 121)	**Primary:** Transfusion avoidance: Hemolysis **Secondary:** % CFB in LDH, FACIT-Fatigue (13-item), and EORTC QLQ-C30; BTH; Hb stabilization, time to first occurrence of LDH normalization; total number of PRBC units transfused; proportion of patients experiencing MAVEs; change in free C5 concentrations	Day 183 Year 1 Year 2
Pegcetacoplan clinical trials					
**PRINCE** Wong 2023 [38]	RCT Open-label International multicentre	Hb ≤ 13.6 g/dL for males and ≤12.0 g/dL for females LDH ≥ 1.5 × ULN	PEG (*n* = 35) vs. Supportive care (excluding ECU/RAV) (*n* = 18)	**Primary:** Hb stabilization; CFB in LDH **Secondary:** Hb response; CFB in ARC; CFB in Hb level; percentage of patients received transfusion and/or decrease in Hb level; transfusion avoidance; PRBC units transfused; FACIT-F scores; EORTC QLQ-C30; ARC normalization	Week 26
**Study 307 OLE ^†^** (including patients from PRINCE) Patriquin 2024 [39]	OLE International multicentre	Participated in a pegcetacoplan clinical trial	PEG (*n* = 50)	NR	Week 4 Week 8 Week 12 Week 24 Week 36 Week 48
Iptacopan clinical trials					
**APPOINT-PNH** De Latour 2024 [40]	Single-arm CT Open-label International multicentre	Hb < 10 g/dL LDH > 1.5 × ULN Platelet count ≥ 30 × 10^9^/L	IPTA (*n* = 40)	**Primary:** Hematological response **Secondary:** Proportion of patients achieving sustained Hb levels ≥ 12 g/dL in the absence of RBC transfusions, transfusion avoidance; CFB in Hb, % CFB in LDH; rate of BTH; CFB in reticulocyte counts; change in FACIT-Fatigue score (13-item), rates of MAVEs	Day 168
Crovalimab clinical trials					
**COMMODORE 2** Roth 2024 [41]	RCT Open-label International multicentre	LDH ≥ 2 × ULN Platelet count ≥ 30,000/mm^3^ White blood cells with granulocyte or monocyte clone size of ≥10%	CROVA (*n* = 135) vs. ECU (*n* = 69)	**Primary:** Hemolysis control; transfusion avoidance **Secondary:** BTH; Hb stabilization, CFB in FACIT-Fatigue (13-item)	Week 5 Week 25
**COMMODORE 3** Liu 2023 [42]	Single-arm CT Open-label China	LDH ≥ 2 × ULN ≥4 transfusions of PRBCs during the 12 months prior to screening	CROVA (*n* = 51)	**Primary:** Hemolysis control; transfusion avoidance **Secondary:** BTH; stabilized Hb; CFB in FACIT-Fatigue	Week 25
Eculizumab biosimilar clinical trials					
**NCT04058158** Jang 2023 [43]	Cross-over RCT Double-blind International multicentre	LDH ≥ 1.5 × ULN ≥10% granulocyte or monocyte clone History of transfusion within 12 months prior to screening	SB12 (ECU biosimilar) switch to ECU (*n* = 25) vs. ECU switch to SB12 (ECU biosimilar) (*n* = 25)	**Primary:** Reduction of hemolysis **Secondary:** Time-course of LDH; number of transfused PRBC units	Week 26 Week 52

^†^ Only data from patients who were enrolled in both the Phase 3 PRINCE trial and the 307 OLE study met the PICOS eligibility criteria. Abbreviations: AE, adverse event; ARC, absolute reticulocyte count; BTH, breakthrough hemolysis; CFB, change from baseline; CI-naïve, complement inhibitor-naïve; CROVA, crovalimab; CT, clinical trial; ECG, electrocardiogram; EORTC QLQ-C30; European Organization For Research and Treatment of Cancer Quality of Life Questionnaire; ECU, eculizumab; FACIT-Fatigue, Functional Assessment of Chronic Illness Therapy-Fatigue; GPI, glucose phosphate isomerase; Hb, hemoglobin; IPTA, iptacopan; LDH, lactate dehydrogenase; MAVE, major adverse vascular event; NR, not reported; OLE, open-label extension; PBO, placebo; PEG, pegcetacoplan; PNH, paroxysmal nocturnal hemoglobinuria; PRBC, packed red blood cell; RAV, ravulizumab; RBC, red blood cells; RCT, randomized controlled trial; ULN, upper limit of normal; USA, United States of America; vs., versus.

**Table 3:** Key study characteristics of clinical trials evaluating patients with CI-experienced PNH and residual anemia.

Clinical Trial Name or Acronym Primary Publication	Study Design; Masking Status	Key Study Inclusion Criteria	Intervention vs. Comparator	Endpoints	Key Analysis Timepoints
**Pegcetacoplan clinical trials**					
**PEGASUS** Hillmen 2021 [44]	RCT Open-label Cross-over International multicentre	Hb < 10.5 g/dL Platelet count > 50 × 10^9^/L Prior treatment with eculizumab	PEG (*n* = 41) versus ECU (*n* = 39)	**Primary:** Change in Hb level from baseline to Week 16 **Secondary:** % of patients who did not require a transfusion during the randomized, controlled period CFB to Week 16 in ARC, LDH level, and FACIT-Fatigue (13-item) score	Week 16
**Study 307 OLE ^†^** (including patients from PEGASUS) Patriquin 2024 [39]	OLE International multicentre	Participated in a pegcetacoplan clinical trial	PEG (*n* = 50)	NR	Week 4 Week 8 Week 12 Week 24 Week 36 Week 48
**Iptacopan clinical trials**					
**APPLY-PNH** De Latour 2024 [40]	RCT Open-label International multicentre	Hb < 10 g/dL Clone size ≥ 10% Prior treatment with eculizumab or ravulizumab	IPTA (*n* = 62) vs. C5i (*n* = 35)	**Primary:** Hematological response **Secondary:** Transfusion avoidance, CFB in Hb level; FACIT-Fatigue (13-item) score; ARC; LDH level; rates of clinical BTH MAVEs; safety	Week 24
**Danicopan clinical trials**					
**ALPHA** Lee 2023 [45]	RCT Double-blind	Hb ≤ 9.5 g/dL Platelet count ≥ 30,000/µL Prior treatment with an approved C5i	DANI + ECU/RAV (*n* = 49) vs. PBO + ECU/RAV (*n* = 29)	**Primary:** CFB in Hb at Week 12 **Secondary:** ≥2 g/dL Hb increase from baseline at Week 12 (or Week 24 in the absence of transfusions) at Week 12 and 24; transfusion avoidance; and CFB in: FACIT-Fatigue at week 12 and week 24; ARC; number of PRBCs transfused at Week 12 and Week 24; bilirubin; PNH RBC clone size; C3; and LDH	Week 12 Week 24

^†^ Only data from patients who were enrolled in both the Phase 3 PEGASUS trial and the 307 OLE study met the PICOS eligibility criteria. Abbreviations: ARC, absolute reticulocyte count; BTH, breakthrough hemolysis; C5i, complement 5 inhibitors; CFB, change from baseline; CI, complement inhibitor; C3, complement 3; DANI, danicopan; ECU, eculizumab; FACIT-Fatigue, Functional Assessment of Chronic Illness Therapy-Fatigue; Hb, hemoglobin; IPTA, iptacopan; LDH, lactate dehydrogenase; MAVE, major adverse vascular event; NR, not reported; OLE, open-label extension; PEG, pegcetacoplan; PNH, paroxysmal nocturnal hemoglobinuria; PRBC, packed red blood cell; RAV, ravulizumab; RBC, red blood cell; RCT, randomized controlled trial;vs., versus.

## Data Availability

The review was not registered, and the protocol of this review was not prepared.

## Supplementary Materials

The following supporting information can be downloaded at: https://www.mdpi.com/article/10.3390/jcm15114217/s1, Table S1: Records included in the systematic literature review; Table S2: Efficacy outcomes reported in clinical trials evaluating patients with CI-naïve PNH; Table S3: FACIT-Fatigue reported in clinical trials evaluating patients with CI-naïve PNH; Table S4: BTH and safety outcomes reported in clinical trials evaluating patients with CI-naïve PNH; Table S5: Efficacy outcomes reported in clinical trials evaluating patients with CI-experienced PNH and residual anemia; Table S6: FACIT-Fatigue reported in clinical trials evaluating patients with CI-experienced PNH and residual anemia; Table S7: BTH and safety outcomes reported in clinical trials evaluating patients with CI-experienced PNH and residual anemia; Table S8: Hemoglobin-related and hemolysis-related definitions provided in trials reporting these outcomes; Table S9: LDH normalization definitions provided in trials reporting this outcome; Table S10: BTH definitions provided in trials reporting this outcome; Table S11: Quality assessment of RCTs using Cochrane RoB 2.0; Table S12: Quality assessment of single-arm trials using a modified version of the CASP checklist; PRISMA checklist.
