# Supplementary material for: Efficacy and Safety of Treatments for Paroxysmal Nocturnal Hemoglobinuria: A Systematic Literature Review
Source: J Clin Med. 2026 May 29;15(11):4217. doi: 10.3390/jcm15114217 (PMC13258451; doi:10.3390/jcm15114217)
Supplement: Supplementary file 1 [file jcm-15-04217-s001.zip › jcm-4252607-supplementary.pdf]

Efficacy and Safety of Treatments for Paroxysmal Nocturnal Hemoglobinuria: A Systematic Literature Review

Shreyans Gandhi [1,*], Isobel Munro [2], Victoria Shodimu [2], Neil Webb [2], Katharina Pannagl [3], Anggie Wiyani [3] and Maria-Magdalena Balp [4]

[1] Department of Haematological Medicine, King's College Hospital NHS Foundation Trust, London SE5 9RS, UK
[2] Source Health Economics, London WC1V 7AA, UK
[3] Novartis Pharmaceuticals UK Ltd., London W12 7FQ, UK
[4] Novartis Pharma AG, 4056 Basel, Switzerland
* Correspondence: shreyans.gandhi@nhs.net

Contents

Supplementary material 2
- Supplementary methods 2
  - Search strings 2
  - Hand-searching 13
  - Included records 15
- Outcomes in the CI-naïve population 26
  - Efficacy outcomes reported in clinical trials 26
  - Health-related quality of life outcomes reported in clinical trials 30
  - Breakthrough hemolysis and safety outcomes reported in clinical trials 31
- Outcomes in the CI-experienced population 34
  - Efficacy outcomes reported in clinical trials 34
  - Health-related quality of life outcomes reported in clinical trials 36
  - Breakthrough hemolysis and safety outcomes reported in clinical trials 37
- Definitions of hemoglobin-related and hemolysis-related efficacy outcomes 38
- Lactate dehydrogenase (LDH) normalization definition 39
- Breakthrough hemolysis (BTH) definition 39
- Risk of bias assessment 41
- PRISMA checklist 43

Supplementary material

Supplementary methods

Search strings

April 2023 original SLR

Database: Embase

Platform: Ovid

Date searched: 20/04/2023

Hits: 931

Study design filter: modified from Scottish Intercollegiate Guidelines Network (SIGN)

#	Searches	Results
1	paroxysmal nocturnal hemoglobinuria/	6333
2	(paroxysmal nocturnal h? emoglobinuria or PNH).ti,ab.	4227
3	or/1-2	7155
4	exp iptacopan/	85
5	(iptacopan or LNP023).ti,ab.	52
6	exp eculizumab/	8893
7	(eculizumab or soliris).ti,ab.	5137
8	complement component C5 inhibitor/	321
9	(anti-C5$ or anti C5$ or antiC5$ or C5 inhibitor).ti,ab.	1507
10	exp ravulizumab/	566
11	(ravulizumab or ultomiris or ALXN1210).ti,ab.	392
12	exp pegcetacoplan/	313
13	(pegcetacoplan or Empaveli or Aspaveli or APL-2).ti,ab.	269
14	exp danicopan/	63
15	(danicopan or ALXN2040 or ACH-0144471).ti,ab.	34
16	exp crovalimab/	81
17	(crovalimab or RG6107 or SKY59).ti,ab.	51
18	exp vemircopan/	6
19	(vemircopan or ALXN2050).ti,ab.	1
20	exp pozelimab/	28
21	(pozelimab or REGN3918).ti,ab.	12
22	exp cemdisiran/	51
23	(cemdisiran or ALN-CC5).ti,ab.	18

#	Searches	Results
24	NM8074.ti,ab.	0
25	ARO-C3.ti,ab.	2
26	KP104.ti,ab.	3
27	CAN-106.ti,ab.	2
28	or/4-27	10422
29	3 and 28	2130
30	exp Clinical Trial/	1837064
31	exp RANDOMIZATION/	99178
32	Single Blind Procedure/	51468
33	Double Blind Procedure/	209423
34	Crossover Procedure/	74891
35	PLACEBO/	401661
36	randomi?ed controlled trial*.tw.	323216
37	rct.tw.	53151
38	(random$ adj3 (allocat$ or assign*)).tw.	233729
39	(((singl$ or doubl$ or treb$ or tripl$) adj (blind$ or mask$)) or (open label adj trial)).tw.	286833
40	placebo$.tw.	366459
41	(clinical adj trial*).tw.	693030
42	((phase 1? or phase 2? or phase 3? or phase 4? or phase i? or phase ii? or phase iii? or phase iv?) adj2 (study or studies or trial*)).tw.	242373
43	or/30-42	2781902
44	Case Study/	97782
45	case report.tw.	529205
46	letter/	1222383
47	Editorial.pt.	771844
48	Letter.pt.	1298932
49	Note.pt.	935407
50	or/44-49	3619408
51	43 not 50	2650189
52	29 and 51	670
53	Clinical study/	162739
54	case control study/	204782
55	Family study/	25765
56	Longitudinal study/	191945
57	Retrospective study/	1452107
58	Prospective study/ not Randomized controlled trials/	858424

#	Searches	Results
59	Cohort analysis/	1022023
60	(Cohort adj (study or studies)).mp.	472881
61	((Case control adj (study or studies)) or case series).tw.	313336
62	(follow up adj (study or studies)).tw.	73499
63	(observational adj (study or studies)).tw.	251497
64	(epidemiologic$ adj (study or studies)).tw.	122143
65	(cross sectional adj (study or studies)).tw.	334650
66	(real adj (world or life)).tw.	173545
67	(RWE or RWD).tw.	2703
68	((single centre or single center or multi centre or multi center or multicentre or multicenter) adj2 experience).tw.	42741
69	audit.tw.	90307
70	(registry or registries).ti.	68193
71	clinical practice.ti.	51879
72	exp registries/	191035
73	exp databases/	550158
74	or/53-73	4760151
75	29 and 74	465
76	52 or 75	976
77	animal/	1604837
78	nonhuman/	7440548
79	exp animal experiment/	3077395
80	exp experimental animal/	825559
81	animal model/	1710167
82	exp rodent/	4011633
83	(rat or rats or mouse or mice).ti.	1608594
84	or/77-83	9920248
85	human/ and 84	2799700
86	84 not 85	7120548
87	76 not 86	963
88	limit 87 to english language	931

Database: MEDLINE, incorporating MEDLINE In-Process & Other non-indexed citations, MEDLINE e-pub ahead-of-print, and MEDLINE Daily

Platform: Ovid

Date searched: 20/04/2023

Hits: 176

Study design filter: modified from Scottish Intercollegiate Guidelines Network (SIGN) (1)

#	Searches	Results
1	Hemoglobinuria, Paroxysmal/	3806
2	(paroxysmal nocturnal h?emoglobinuria or PNH).ti,ab.	3807
3	or/1-2	4971
4	(iptacopan or LNP023).mp.	10
5	(eculizumab or soliris).mp.	2503
6	Complement C5/	2725
7	(anti-C5$ or anti C5$ or antiC5$ or C5 inhibitor).ti,ab.	795
8	(ravulizumab or ultomiris or ALXN1210).mp.	144
9	(pegcetacoplan or Empaveli or Aspaveli or APL-2).mp.	78
10	(danicopan or ALXN2040 or ACH-0144471).mp.	10
11	(crovalimab or RG6107 or SKY59).mp.	16
12	(vemircopan or ALXN2050).mp.	0
13	(pozelimab or REGN3918).mp.	3
14	(cemdisiran or ALN-CC5).mp.	7
15	NM8074.ti,ab.	0
16	ARO-C3.ti,ab.	2
17	KP104.ti,ab.	1
18	CAN-106.ti,ab.	0
19	or/4-18	5453
20	3 and 19	659
21	Randomized Controlled Trials as Topic/	161626
22	randomized controlled trial/	591095
23	Random Allocation/	106922
24	Double Blind Method/	174906
25	Single Blind Method/	32639
26	exp clinical trial/	968213
27	exp Clinical Trials as topic/	381743
28	PLACEBOS/	35926
29	(clinical adj trial*).tw.	470778
30	(((singl$ or doubl$ or treb$ or tripl$) adj (blind$ or mask$)) or (open label adj trial)).tw.	199641
31	placebo$.tw.	245172
32	(random$ adj2 allocat$).tw.	43205

#	Searches	Results
33	randomi?ed controlled trial*.tw.	242682
34	rct.tw.	30641
35	((phase 1? or phase 2? or phase 3? or phase 4? or phase i? or phase ii? or phase iii? or phase iv?) adj2 (study or studies or trial*)).tw.	113851
36	or/21-35	1810491
37	case report.tw.	390655
38	letter/	1213612
39	historical article/	369199
40	or/37-39	1954695
41	36 not 40	1768855
42	20 and 41	116
43	Epidemiologic studies/	9307
44	exp case control studies/	1407950
45	exp cohort studies/	2470779
46	Cross-sectional studies/	463648
47	multicenter study/ not exp clinical trial/	196239
48	Case control.tw.	152032
49	(cohort adj (study or studies or analy*)).tw.	318169
50	(follow up adj (study or studies)).tw.	55821
51	(observational adj (study or studies)).tw.	157334
52	Longitudinal.tw.	316984
53	Retrospective.tw.	729085
54	Cross sectional.tw.	501500
55	(epidemiologic$ adj (study or analy*)).tw.	29623
56	(real adj (world or life)).tw.	100281
57	(RWE or RWD).tw.	1010
58	((single centre or single center or multi centre or multi center or multicentre or multicenter) adj2 experience).tw.	18128
59	audit.tw.	40050
60	(registry or registries).ti.	37209
61	clinical practice.ti.	37405
62	Databases, Factual/	97657
63	exp registries/	116624
64	or/43-63	4065287
65	20 and 64	95
66	42 or 65	192

#	Searches	Results
67	animals/	7264603
68	exp animals, laboratory/	949254
69	exp animal experimentation/	10311
70	exp models, animal/	639017
71	exp rodentia/	3528145
72	(rat or rats or mouse or mice).ti.	1432033
73	or/67-72	7375521
74	humans/ and 73	2192165
75	73 not 74	5183356
76	66 not 75	192
77	limit 76 to english language	176

Database: Cochrane library

Platform: Ovid

Date searched: 20/04/2023

Hits: 249

Study design filter: modified from Scottish Intercollegiate Guidelines Network (SIGN) (1)

#	Searches	Results
1	Hemoglobinuria, Paroxysmal/	72
2	(paroxysmal nocturnal h?emoglobinuria or PNH).ti,ab.	318
3	or/1-2	326
4	(iptacopan or LNP023).mp.	29
5	(eculizumab or soliris).mp.	454
6	Complement C5/	41
7	(anti-C5$ or anti C5$ or antiC5$ or C5 inhibitor).ti,ab.	158
8	(ravulizumab or ultomiris or ALXN1210).mp.	153
9	(pegcetacoplan or Empaveli or Aspaveli or APL-2).mp.	139
10	(danicopan or ALXN2040 or ACH-0144471).mp.	20
11	(crovalimab or RG6107 or SKY59).mp.	23
12	(vemircopan or ALXN2050).mp.	8
13	(pozelimab or REGN3918).mp.	13
14	(cemdisiran or ALN-CC5).mp.	18
15	NM8074.ti,ab.	1

#	Searches	Results
16	ARO-C3.ti,ab.	1
17	KP104.ti,ab.	4
18	CAN-106.ti,ab.	0
19	or/4-18	712
20	3 and 19	253
21	limit 20 to english language [Limit not valid in CDSR; records were retained]	249

September 2024 SLR update

Database: Embase

Platform: Ovid

Date searched: 18/09/2024

Hits: 157

Study design filter: modified from Scottish Intercollegiate Guidelines Network (SIGN) (1)

#	Searches	Results
1	paroxysmal nocturnal hemoglobinuria/	6902
2	(paroxysmal nocturnal h?emoglobinuria or PNH).ti,ab.	6353
3	or/1-2	8150
4	exp iptacopan/	210
5	(iptacopan or fabhalta or LNP023).ti,ab.	135
6	exp eculizumab/	10341
7	(eculizumab or soliris).ti,ab.	5959
8	complement component C5 inhibitor/	406
9	(anti-C5$ or anti C5$ or antiC5$ or C5 inhibitor).ti,ab.	1732
10	exp ravulizumab/	917
11	(ravulizumab or ultomiris or ALXN1210).ti,ab.	592
12	exp pegcetacoplan/	548
13	(pegcetacoplan or Empaveli or Aspaveli or APL-2).ti,ab.	416
14	exp danicopan/	109
15	(danicopan or ALXN2040 or ACH-0144471).ti,ab.	52
16	exp crovalimab/	134
17	(crovalimab or RG6107 or SKY59).ti,ab.	84

#	Searches	Results
18	(ruxoprubart or NM8074).ti,ab.	0
19	ARO-C3.ti,ab.	1
20	KP104.ti,ab.	6
21	(omoprubart or CAN-106).ti,ab.	2
22	or/4-21	12272
23	3 and 22	2482
24	exp Clinical Trial/	1950347
25	exp RANDOMIZATION/	100490
26	Single Blind Procedure/	56537
27	Double Blind Procedure/	224377
28	Crossover Procedure/	79879
29	PLACEBO/	418656
30	randomi?ed controlled trial*.tw.	360124
31	rct.tw.	59772
32	(random$ adj3 (allocat$ or assign*)).tw.	252264
33	(((singl$ or doubl$ or treb$ or tripl$) adj (blind$ or mask$)) or open label).tw.	401947
34	placebo$.tw.	386560
35	(clinical adj trial*).tw.	761163
36	((phase 1? or phase 2? or phase 3? or phase 4? or phase i? or phase ii? or phase iii? or phase iv?) adj2 (study or studies or trial*)).tw.	264375
37	or/24-36	2989052
38	Case Study/	103459
39	case report.tw.	587855
40	letter/	1258384
41	Editorial.pt.	824103
42	Letter.pt.	1344521
43	Note.pt.	1003201
44	or/38-43	3848067
45	37 not 44	2851133
46	23 and 45	814
47	animal/	1681696
48	nonhuman/	7885235
49	exp animal experiment/	3249866

#	Searches	Results
50	exp experimental animal/	870432
51	animal model/	1838407
52	exp rodent/	4210701
53	(rat or rats or mouse or mice).ti.	1661202
54	or/47-53	10450067
55	human/ and 54	3014998
56	54 not 55	7435069
57	46 not 56	803
58	limit 57 to dd=20230420-20240918	101
59	limit 57 to dc=20230420-20240918	158
60	or/58-59	158
61	limit 60 to english language	157

Database: MEDLINE, incorporating MEDLINE In-Process & Other non-indexed citations, MEDLINE e-pub ahead-of-print, and MEDLINE Daily

Platform: Ovid

Date searched: 18/09/2024

Hits: 47

Study design filter: modified from Scottish Intercollegiate Guidelines Network (SIGN)

#	Searches	Results
1	Hemoglobinuria, Paroxysmal/	3943
2	(paroxysmal nocturnal h?emoglobinuria or PNH).ti,ab.	4028
3	or/1-2	5207
4	(iptacopan or fabhalta or LNP023).mp.	45
5	(eculizumab or soliris).mp.	2872
6	Complement C5/	2776
7	(anti-C5$ or anti C5$ or antiC5$ or C5 inhibitor).ti,ab.	883
8	(ravulizumab or ultomiris or ALXN1210).mp.	249
9	(pegcetacoplan or Empaveli or Aspaveli or APL-2).mp.	177
10	(danicopan or ALXN2040 or ACH-0144471).mp.	26
11	(crovalimab or RG6107 or SKY59).mp.	28

#	Searches	Results
1	Hemoglobinuria, Paroxysmal/	3943
12	(ruxoprubart or NM8074).ti,ab.	0
13	ARO-C3.ti,ab.	1
14	KP104.ti,ab.	1
15	(omoprubart or CAN-106).ti,ab.	0
16	or/4-15	5982
17	3 and 16	770
18	Randomized Controlled Trials as Topic/	173924
19	randomized controlled trial/	621730
20	Random Allocation/	107580
21	Double Blind Method/	180416
22	Single Blind Method/	33998
23	exp clinical trial/	1004407
24	exp Clinical Trials as topic/	397306
25	PLACEBOS/	35994
26	(clinical adj trial*).tw.	525674
27	(((singl$ or doubl$ or treb$ or tripl$) adj (blind$ or mask$)) or open label).tw.	262030
28	placebo$.tw.	260002
29	(random$ adj2 allocat$).tw.	47431
30	randomi?ed controlled trial*.tw.	278618
31	rct.tw.	35511
32	((phase 1? or phase 2? or phase 3? or phase 4? or phase i? or phase ii? or phase iii? or phase iv?) adj2 (study or studies or trial*)).tw.	124047
33	or/18-32	1935733
34	case report.tw.	438723
35	letter/	1273554
36	historical article/	371859
37	or/34-36	2063335
38	33 not 37	1891608
39	17 and 38	162
40	animals/	7510613
41	exp animals, laboratory/	973080
42	exp animal experimentation/	10577

#	Searches	Results
1	Hemoglobinuria, Paroxysmal/	3943
43	exp models, animal/	661228
44	exp rodentia/	3643665
45	(rat or rats or mouse or mice).ti.	1479613
46	or/40-45	7634523
47	humans/ and 46	2293326
48	46 not 47	5341197
49	39 not 48	162
50	limit 49 to dt=20230420-20240918	42
51	limit 49 to ed=20230420-20240918	33
52	or/50-51	47
53	limit 52 to english language	47

Database: Cochrane library

Platform: Ovid

Date searched: 18/09/2024

Hits: 61

Study design filter: N/A

#	Searches	Results
1	Hemoglobinuria, Paroxysmal/	98
2	(paroxysmal nocturnal h?emoglobinuria or PNH).ti,ab.	396
3	or/1-2	411
4	(iptacopan or fabhalta or LNP023).mp.	69
5	(eculizumab or soliris).mp.	525
6	Complement C5/	51
7	(anti-C5$ or anti C5$ or antiC5$ or C5 inhibitor).ti,ab.	203
8	(ravulizumab or ultomiris or ALXN1210).mp.	217
9	(pegcetacoplan or Empaveli or Aspaveli or APL-2).mp.	184
10	(danicopan or ALXN2040 or ACH-0144471).mp.	23
11	(crovalimab or RG6107 or SKY59).mp.	49
12	(ruxoprubart or NM8074).ti,ab.	2
13	ARO-C3.ti,ab.	1

#	Searches	Results
14	KP104.ti,ab.	6
15	(omoprubart or CAN-106).ti,ab.	0
16	or/4-15	892
17	3 and 16	316
18	limit 17 to yr="2023 -Current"	61

Hand-searching

The following conferences held from January 2020 to September 2024 were searched:

- American Society of Hematology (ASH)
- European Haematology Association (EHA)
- European Society for Blood and Marrow Transplantation (EBMT)
- International Society for Pharmacoeconomics and Outcomes Research (ISPOR).

Submission documents on the following health technology assessment agency websites, published from January 2020 to September 2024, were reviewed for relevant clinical data:

- England: National Institute for Health and Care Excellence (NICE)
- Scotland: Scottish Medicines Consortium (SMC)
- Wales: All Wales Medicines Strategy Group (AWMSG)
- Ireland: National Centre for Pharmacoeconomics (NCPE)
- Australia: Pharmaceutical Benefits Advisory Committee (PBAC)
- Canada: Canadian Agency for Drugs and Technologies in Health (CADTH)
- France: Haute Autorité de Santé (HAS)
- Germany:
    - German Institute for Quality and Efficiency in Health Care (IQWiG)
    - Gemeinsamer Bundesausschuss (The Federal Joint Committee [G-BA])
- United States of America: Institute for Clinical and Economic Review.

To obtain details of potentially relevant published and ongoing trials, the following clinical trial registry databases were accessed:

- World Health Organization International Clinical Trials Registry Platform (WHO ICTRP: https://www.who.int/ictrp/search/en/)
- United States National Institutes of Health (NIH) trial registry & results database (https://clinicaltrials.gov/).

The following additional databases were hand-searched:

- European Medicines Agency (EMA: https://www.ema.europa.eu/en)
- United States (US) Food & Drug Administration (FDA: https://www.fda.gov/).

Included records

Supplementary Table S1: Records included in the systematic literature review

Trial name	Primary or linked records?	Authors	Title	Published year
**Clinical trials – complement inhibitor-naïve population**				
**AEGIS**	Primary	Kanakura Y.; Ohyashiki K.; Shichishima T. et al	Safety and efficacy of the terminal complement inhibitor eculizumab in Japanese patients with paroxysmal nocturnal hemoglobinuria: The AEGIS Clinical Trial	2011
	Linked	Kanakura Y.; Ohyashiki K.; Shichishima T. et al	Long-term efficacy and safety of eculizumab in Japanese patients with PNH: AEGIS trial	2013
**APPOINT-PNH**	Primary	de Latour R.P.; Roth A.; Kulasekararaj A.G.; Han B. et al	Oral Iptacopan Monotherapy in Paroxysmal Nocturnal Hemoglobinuria	2024
	Linked	Novartis	APPOINT-PNH CSR. A multicenter, single-arm, open-label trial to evaluate efficacy and safety of oral twice daily iptacopan in adult PNH patients who are naive to complement inhibitor therapy.	2022
	Linked	Novartis	APPOINT-PNH CSR (Final analysis): A multicenter, single-arm, open-label trial to evaluate efficacy and safety of oral, twice daily iptacopan in adult PNH patients who are naive to complement inhibitor therapy	2023
	Linked	de Latour 2023	Substantial Increases in Paroxysmal Nocturnal Hemoglobinuria (PNH) Red Blood Cell Clone Size With Oral Iptacopan Monotherapy Confirms Control of Hemolysis in Complement Inhibitor-Naive PNH Patients	2023
**COMMODORE 2**	Primary	Roth, A; He, G; Tong, H; Lin, Z. et al	Phase 3 randomized COMMODORE 2 trial: Crovalimab versus eculizumab in patients with paroxysmal nocturnal hemoglobinuria naive to complement inhibition.	2024
	Linked	Lundberg P.; de la Iglesia S.; Kelly R.J.; Kulasekararaj A. et al	Biomarker Analyses in Patients with Paroxysmal Nocturnal Hemoglobinuria (PNH) Treated with Crovalimab and Eculizumab: Results from the Phase III Randomized COMMODORE 2 Trial	2023
	Linked	Panse J.; Cermak J.; Kyselova O.; Gotoh A. et al	Patient-Reported Outcomes (PROs) in Patients with Paroxysmal Nocturnal Hemoglobinuria (PNH) Treated with Crovalimab and Eculizumab: Results from the Phase III Randomized COMMODORE 2 and COMMODORE 1 Trials	2023
	Linked	European Medicines Agency (EMA)	EPAR: piasky	2024
**COMMODORE 3**	Primary	Liu H.; Xia L.; Weng J.; Zhang F et al	Efficacy and safety of the C5 inhibitor crovalimab in complement inhibitor-naive patients with PNH (COMMODORE 3): A multicenter, Phase 3, single-arm study	2023

Trial name	Primary or linked records?	Authors	Title	Published year
	Linked	NCT record	A Study Evaluating the Efficacy, Safety, Pharmacokinetics and Pharmacodynamics of Crovalimab in Participants With Paroxysmal Nocturnal Hemoglobinuria (PNH) Not Previously Treated With Complement Inhibition (COMMODORE 3)	2024
**Eculizumab extension study**	Primary	Hillmen P.; Muus P.; Roth A. et al	Long-term safety and efficacy of sustained eculizumab treatment in patients with paroxysmal nocturnal haemoglobinuria	2013
	Linked	Brodsky R.A.; De Castro C.; Schrezenmeier H. et al	Long term safety and efficacy of sustained eculizumab treatment in patients with paroxysmal nocturnal hemoglobinuria (PNH)	2010
	Linked	Hillmen P.; Muus P.; Duhrsen U. et al	Effect of the complement inhibitor eculizumab on thromboembolism in patients with paroxysmal nocturnal hemoglobinuria	2007
	Linked	Hillmen P.; Risitano A.; Schrezenmeier H. et al	Long term outcomes in patients with paroxysmal nocturnal hemoglobinuria (PNH) with sustained eculizumab treatment	2011
	Linked	Schubert J.	Blockade of terminal complement cascade. Clinical effects of eculizumab in patients with paroxysmal nocturnal haemoglobinuria (PNH)	2009
**PRINCE**	Primary	Wong, R. S. M., Navarro-Cabrera, J. R., Comia et al	Pegcetacoplan controls hemolysis in complement inhibitor–naïve patients with paroxysmal nocturnal hemoglobinuria	2023
	Linked	Wong, R., Al-Adhami, M., Savage, J. et al	Pegcetacoplan rapidly stabilizes complement inhibitor naive patients with paroxysmal nocturnal hemoglobinuria experiencing hemolysis with acute hemoglobin decreases; prince trial post hoc analysis	2022
	Linked	Gomez-Almaguer, D., Wong, R., Dumagay, T. et al	Effect of pegcetacoplan on quality of life in complement-inhibitor naive patients with paroxysmal nocturnal hemoglobinuria: results from the phase 3 prince study	2022
	Linked	NCT record	A Study to Evaluate the Efficacy and Safety of Pegcetacoplan in Patients With PNH	2022
	Linked	Mulherin, B.P; Yeh, M.; Al-Adhami, M. et al	Normalization of Hemoglobin, Lactate Dehydrogenase, and Fatigue in Patients with Paroxysmal Nocturnal Hemoglobinuria Treated with Pegcetacoplan.	2024
	Linked	Panse J; Daguindau N; Okuyama S et al	Improvements in hematologic markers and decreases in fatigue with pegcetacoplan for patients with paroxysmal nocturnal hemoglobinuria and mild or moderate anemia (hemoglobin ≥10 g/dL) who had received eculizumab or were naive to complement inhibitors	2024
**SHEPHERD**	Primary	Brodsky R.A.; Young N.S.; Antonioli E. et al	Multicenter phase 3 study of the complement inhibitor eculizumab for the treatment of patients with paroxysmal nocturnal hemoglobinuria	2008

Trial name	Primary or linked records?	Authors	Title	Published year
	Linked	Schubert J.; Hillmen P.; Roth A. et al	Eculizumab, a terminal complement inhibitor, improves anaemia in patients with paroxysmal nocturnal haemoglobinuria	2008
**Study 201**	Primary	Roth A.; Rottinghaus S.T.; Hill A. et al	Ravulizumab (ALXN1210) in patients with paroxysmal nocturnal hemoglobinuria: Results of 2 phase 1b/2 studies	2018
	Linked	NCT record	A Phase 2, Open-Label, Multiple Ascending Dose Study to Evaluate the Efficacy, Safety, Tolerability, Immunogenicity, Pharmacokinetics, and Pharmacodynamics of ALXN1210 Administered Intravenously to Patients with Paroxysmal Nocturnal Hemoglobinuria	2015
**Study 301**	Primary	Lee J.W.; de Fontbrune F.S.; Lee L.W.L. et al	Ravulizumab (ALXN1210) vs eculizumab in adult patients with PNH naive to complement inhibitors: The 301 study	2019
	Linked	De Latour R.P.; Szer J.; Kulasekararaj A. et al	Efficacy and safety of ravulizumab in older patients aged >65 years with paroxysmal nocturnal hemoglobinuria in the 301 and 302 phase 3 extension studies	2020
	Linked	Peffault De Latour R.; Hill A.; Fureder W. et al	Ravulizumab reduces the risk of thrombosis in adult patients with paroxysmal nocturnal hemoglobinuria and high disease activity: 2-Year data from a phase 3, open-label study	2021
	Linked	Ishiyama K.; Nakao S.; Usuki K. et al	Results from multinational phase 3 studies of ravulizumab (ALXN1210) versus eculizumab in adults with paroxysmal nocturnal hemoglobinuria: subgroup analysis of Japanese patients	2020
	Linked	Kulasekararaj A.G.; Griffin M.; Langemeijer S. et al	Long-term safety and efficacy of ravulizumab in patients with paroxysmal nocturnal hemoglobinuria: 2-year results from two pivotal phase 3 studies	2022
	Linked	Risitano A.; Jang J.-H.; Gyeong-Won L. et al	Transfusion requirements in adult patients with paroxysmal nocturnal hemoglobinuria with or without a history of bone marrow disorder receiving ravulizumab and eculizumab: Results from a phase 3 non-inferiority study extension	2020
	Linked	Schrezenmeier H.; Kulasekararaj A.; Mitchell L. et al	One-year efficacy and safety of ravulizumab in adults with paroxysmal nocturnal hemoglobinuria naive to complement inhibitor therapy: open-label extension of a randomized study	2020
	Linked	Schrezenmeier H.; Lee J.W.; Hill A. et al	Efficacy and safety of concomitant use of ravulizumab and IST in patients with paroxysmal nocturnal hemoglobinuria up to 52 weeks	2020
	Linked	NCT record	ALXN1210 (Ravulizumab) Versus Eculizumab in Complement Inhibitor Treatment-Naive Adult Participants With Paroxysmal Nocturnal Hemoglobinuria (PNH)	2016

Trial name	Primary or linked records?	Authors	Title	Published year
	Linked	EMA	Ultomiris: EPAR 2019	2019
	Linked	Kulasekararaj A.; Schrezenmeier H.; Usuki K.; Kulagin A et al	Ravulizumab Provides Durable Control of Intravascular Hemolysis and Improves Survival in Patients with Paroxysmal Nocturnal Hemoglobinuria: Long-Term Follow-up of Study 301 and Comparisons with Patients of the International PNH Registry	2023
**Study 307 OLE[†]**	Primary	Patriquin, C.J.; Bogdanovic, A.; Griffin, M.; Kelly, R.J; Maciejewski, J.P; Mulherin, B; Peffault de Latour, R.; Roth, A.; Selvaratnam, V.; Szer, J.; Al-Adhami, M.; Horneff, R.; Tan, L.; Yeh, M.; Panse, J.	Safety and Efficacy of Pegcetacoplan in Adult Patients with Paroxysmal Nocturnal Hemoglobinuria over 48 Weeks: 307 Open-Label Extension Study.	2024
**TRIUMPH**	Primary	Hillmen P.; Young N.S.; Schubert J. et al	The complement inhibitor eculizumab in paroxysmal nocturnal hemoglobinuria	2006
	Linked	Schubert J.; Hillmen P.; Roth A. et al	Eculizumab, a terminal complement inhibitor, improves anaemia in patients with paroxysmal nocturnal haemoglobinuria	2008
—	Primary	Risitano A.M.; Kulasekararaj A.G.; Lee J.W. et al	Danicopan: An oral complement factor D inhibitor for paroxysmal nocturnal hemoglobinuria	2021
—	Primary	Hillmen P.; Hall C.; Marsh J.C.W. et al	Effect of Eculizumab on Hemolysis and Transfusion Requirements in Patients with Paroxysmal Nocturnal Hemoglobinuria	2004
	Linked	Hill A.; Hillmen P.; Richards S.J. et al	Sustained response and long-term safety of eculizumab in paroxysmal nocturnal hemoglobinuria	2005
—	Primary	Jang J.H.; Wong L.; Ko B.-S. et al	Iptacopan monotherapy in patients with paroxysmal nocturnal hemoglobinuria: a 2-cohort open-label proof-of-concept study	2022
—	Primary	Jang J.H.; Gomez R.D.; Bumbea H. et al	A phase III, randomised, double-blind, multi-national clinical trial comparing SB12 (proposed eculizumab biosimilar) and reference eculizumab in patients with paroxysmal nocturnal haemoglobinuria	2023
	Linked	Jang J.H.; Lim S.M.; Tomuleasa C.; Oliynyk H. et al	Efficacy of SB12 (Eculizumab Biosimilar) in Asian and Non-Asian Patients with Paroxysmal Nocturnal Hemoglobinuria: Subgroup Analysis of a Global Phase III Randomized Controlled Trial	2023
	Linked	EMA	Epysqli: EPAR 2023	2023

Trial name	Primary or linked records?	Authors	Title	Published year
–	Primary	Zhang F.; Zhang L.; Yang C.; Wang C. et al	KP104, a Bifunctional C5 Antibody/Factor H Fusion Protein, Effectively Controls Both Intravascular and Extravascular Hemolysis: Interim Results from a Phase 2 Study in Complement Inhibitor-Naive PNH Patients	2023
**Clinical trials – complement inhibitor-experienced population**				
**ALPHA**	Primary	Lee, J.W.; Griffin, M.; Kim, J.S. et al	Addition of danicopan to ravulizumab or eculizumab in patients with paroxysmal nocturnal haemoglobinuria and clinically significant extravascular haemolysis (ALPHA): a double-blind, randomised, phase 3 trial	2023
	Linked	Piatek C.I.; Lee J.-W.; Griffin M. et al	Patient-Reported Outcomes: Danicopan As Add-on Therapy to Ravulizumab or Eculizumab Versus Placebo in Patients with Paroxysmal Nocturnal Hemoglobinuria and Clinically Significant Extravascular Hemolysis	2023
	Linked	Kulasekararaj A.; Griffin M.; Piatek C.I. et al	Danicopan As Add-on Therapy to Ravulizumab or Eculizumab Versus Placebo in Patients with Paroxysmal Nocturnal Hemoglobinuria and Clinically Significant Extravascular Hemolysis: Phase 3 Long-Term Data	2023
**APPLY-PNH**	Primary	de Latour R.P.; Roth A.; Kulasekararaj A.G.; Han B. et al	Oral Iptacopan Monotherapy in Paroxysmal Nocturnal Hemoglobinuria	2024
	Linked	Novartis	APPLY-PNH CSR. A randomized, multicenter, active-comparator controlled, open-label trial to evaluate efficacy and safety of oral, twice daily LNP023 in adult patients with PNH and residual anemia, despite treatment with an intravenous anti-C5 antibody	2021
	Linked	Novartis	APPLY-PNH CSR (Final analysis). A randomized, multicenter, active-comparator controlled, open-label trial to evaluate efficacy and safety of oral, twice daily LNP023 in adult patients with PNH and residual anemia, despite treatment with an intravenous anti-C5 antibody	2023
**PEGASUS**	Primary	Hillmen P.; Szer J.; Weitz I. et al	Pegcetacoplan versus eculizumab in paroxysmal nocturnal hemoglobinuria	2021
	Linked	de Latour R.P.; Szer J.; Weitz I.C. et al	Pegcetacoplan versus eculizumab in patients with paroxysmal nocturnal haemoglobinuria (PEGASUS): 48-week follow-up of a randomised, open-label, phase 3, active-comparator, controlled trial	2022
	Linked	Cella D.; Sarda S.P.; Hsieh R. et al	Changes in hemoglobin and clinical outcomes drive improvements in fatigue, quality of life, and physical function in patients with paroxysmal nocturnal hemoglobinuria: post hoc analyses from the phase III PEGASUS study	2022

Trial name	Primary or linked records?	Authors	Title	Published year
	Linked	De Latour R.P.; De Castro C.M.D.; Szer J. et al	Long-term effects in subgroups of patients with paroxysmal nocturnal hemoglobinuria treated with pegcetacoplan versus eculizumab: 48-week analysis of pegasus phase 3 trial	2021
	Linked	Panse J.; Hochsmann B.; Griffin M. et al	Effect of pegcetacoplan on quality of life in patients with paroxysmal nocturnal hemoglobinuria: Week 48 of PEGASUS phase 3 trial comparing pegcetacoplan to eculizumab	2021
	Linked	NCT record	A Phase III Study to Evaluate the Efficacy and Safety of APL-2 in Patients With PNH	2019
	Linked	Desai D.; Panse J.; Daguindau N. et al	MDS-112 Normalization of Hematologic and Health-Related Quality of Life Markers in Patients With Paroxysmal Nocturnal Hemoglobinuria Treated With Pegcetacoplan and Baseline Hemoglobin at or Above 10 g/dL	2022
	Linked	Mulherin B.; Yeh M.; Al-Adhami M. et al	Hemoglobin, lactate dehydrogenase, and facit-fatigue normalization rates in patients treated with pegcetacoplan: Results from the pegasus and prince phase 3 clinical trials	2022
	Linked	Panse J.; Daguindau N.; Sasaki S.O. et al	Post hoc analysis of the effect of pegcetacoplan treatment of patients with paroxysmal nocturnal hemoglobinuria and baseline hemoglobin levels greater than 10 grams per deciliter	2021
	Linked	EMA	Aspaveli: EPAR 2021	2021
	Linked	Mulherin, B.P; Yeh, M.; Al-Adhami, M. et al	Normalization of Hemoglobin, Lactate Dehydrogenase, and Fatigue in Patients with Paroxysmal Nocturnal Hemoglobinuria Treated with Pegcetacoplan.	2024
	Linked	Panse J; Daguindau N; Okuyama S et al	Improvements in hematologic markers and decreases in fatigue with pegcetacoplan for patients with paroxysmal nocturnal hemoglobinuria and mild or moderate anemia (hemoglobin ≥10 g/dL) who had received eculizumab or were naive to complement inhibitors	2024
	Linked	Nishimori H.; Nakazawa H.; Tamura S et al	Efficacy, Safety, and Quality of Life of Pegcetacoplan in Japanese Patients with Paroxysmal Nocturnal Hemoglobinuria Treated within the Phase 3 PEGASUS Trial	2024
**Study 307 OLE**[†]	Primary	Patriquin, C.J.; Bogdanovic, A.; Griffin, M et al	Safety and Efficacy of Pegcetacoplan in Adult Patients with Paroxysmal Nocturnal Hemoglobinuria over 48 Weeks: 307 Open-Label Extension Study.	2024
—	Primary	Risitano A.M.; Roth A.; Soret J. et al	Addition of iptacopan, an oral factor B inhibitor, to eculizumab in patients with paroxysmal nocturnal haemoglobinuria and active haemolysis: an open-label, single-arm, phase 2, proof-of-concept trial	2021

Trial name	Primary or linked records?	Authors	Title	Published year
	Linked	NCT record	Study of Safety, Efficacy, Tolerability, Pharmacokinetics and Pharmacodynamics of LNP023 in in Patients With Paroxysmal Nocturnal Hemoglobinuria (PNH)	2024
—	Primary	Kulasekararaj A.G.; Risitano A.M.; Maciejewski J.P. et al	Phase 2 study of danicopan in patients with paroxysmal nocturnal hemoglobinuria with an inadequate response to eculizumab	2021
	Linked	Kulasekararaj A.; Risitano A.; MacIejewski J. et al	A phase 2 open-label study of danicopan (ACH-0144471) in patients with paroxysmal nocturnal haemoglobinuria who have an inadequate response to eculizumab monotherapy	2020
	Linked	Kulasekararaj A.; Risitano A.; Maciejewski J. et al	Effects of oral, factor D inhibitor danicopan on transfusion rates in transfusion dependent paroxysmal nocturnal hemoglobinuria (PNH) patients with a sub-optimal response to eculizumab: Phase 2 study	2020
**Large observational studies (n>100) or registries**				
**APPEX**	NA	Novartis	APPEX CSR	2023
**French Society of Hematology Network**	NA	Loschi M.; Porcher R.; Barraco F. et al	Impact of eculizumab treatment on paroxysmal nocturnal hemoglobinuria: A treatment versus no-treatment study	2016
**International PNH Registry**	NA	Almeida A.M.; Bedrosian C.; Cole A. et al	Clinical benefit of eculizumab in patients with no transfusion history in the International Paroxysmal Nocturnal Haemoglobinuria Registry	2017
	NA	Hill A.; Roeth A.; Socie G. et al	Interim analysis of safety outcomes during treatment with eculizumab: Results from the international paroxysmal nocturnal hemoglobinuria registry	2017
	NA	Griffin M.; Terriou L.; Patriquin C.J. et al	Long-term Survival Benefit of Eculizumab Treatment in Patients With Paroxysmal Nocturnal Haemoglobinuria: Data From the International PNH Registry	2022
	NA	Hill A.; De Latour R.P.; Kulasekararaj A.G. et al	Concomitant Immunosuppressive Therapy and Eculizumab Use in Patients with Paroxysmal Nocturnal Hemoglobinuria: An International PNH Registry Analysis	2023
	NA	Cella D.; Johansson P.; Ueda Y. et al	Clinically important difference for the facit-fatigue scale in paroxysmal nocturnal hemoglobinuria: A derivation from international PNH registry patient data	2021
	NA	Hochsmann B.; de Fontbrune F.S.; Lee J.W. et al	Effect of eculizumab treatment in patients with paroxysmal nocturnal hemoglobinuria with or without high disease activity: Real-world findings from the International Paroxysmal Nocturnal Hemoglobinuria Registry	2022
	NA	Roth A.; Araten D.J.; Larratt L. et al	Beneficial effects of eculizumab regardless of prior transfusions or bone marrow disease: Results of the International Paroxysmal Nocturnal Hemoglobinuria Registry	2020

Trial name	Primary or linked records?	Authors	Title	Published year
	NA	Roth A.; Raiser C.; Panse J. et al	Changes in the burden of disease in treated and untreated patients in the German cohort of the International Registry of Paroxysmal Nocturnal Hemoglobinuria (PNH)-a 2020 Update	2020
	NA	Lee J.W.; Ueda Y.; Gustovic P.et al	Effectiveness of eculizumab treatment in patients aged >= 65 years with paroxysmal nocturnal hemoglobinuria (PNH): Results from the international PNH registry	2020
	NA	Muus P.; Langemeijer S.; Hochsmann B. et al	Patient-reported outcomes and healthcare resource utilization before and during treatment with eculizumab: Results from the international paroxysmal nocturnal hemoglobinuria registry	2017
	NA	Lisukov I.; Kulagin A.; Maschan A. et al	Effect of eculizumab on physician-reported symptoms in the russian cohort of the paroxysmal nocturnal hemoglobinuria (PNH) international registry	2014
	NA	Roth A.; Herich-Terhurne D.; Alashkar F. et al	Thrombotic complications in a single-centre German patient cohort of the International Paroxysmal Nocturnal Hemoglobinuria Registry	2014
	NA	Villegas A.; Gaya A.; Ojeda E. et al	Periodic evaluation of the clone size is mandatory in PNH: Study of the spanish cohort of the international PNH registry	2013
	NA	EMA	Soliris: EPAR 2023	2023
**Korean PNH Registry**	NA	Choi C.W.; Jang J.H.; Kim J.S. et al	Efficacy of eculizumab in paroxysmal nocturnal hemoglobinuria patients with or without aplastic anemia: Prospective study of a Korean PNH cohort	2017
**OPERA**	NA	Fishman J., Min J., Arnett, L., Shenoy A. et al	Early Hemoglobin and Quality-of-Life Trends from OPERA: A Real-World Study of Pegcetacoplan Treatment in US Adults with Paroxysmal Nocturnal Hemoglobinuria	2023
**PMS Database**	NA	Ikezoe T.; Noji H.; Ueda Y. et al	Long-term follow-up of patients with paroxysmal nocturnal hemoglobinuria treated with eculizumab: post-marketing surveillance in Japan	2022
**PNH Swiss Soliris and Ultomiris Reimbursement Registry**	NA	Rovo A.; Simeon L.; Gavillet M. et al	Real-world evidence of safety and effectiveness of eculizumab and switch to ravulizumab in a swiss patient population with paroxysmal nocturnal hemoglobinuria	2022
**Spanish PNH Registry**	NA	De La Iglesia S.; Morado M.; Arrizabalaga B. et al	Paroxysmal nocturnal hemoglobinuria and aplastic anaemia - Data from the Spanish PNH registry	2017
	NA	Villegas A.M.; Nunez R.; Gaya A. et al	Acute and chronic renal failure in a patient cohort from the Spanish PNH registry	2015

Trial name	Primary or linked records?	Authors	Title	Published year
TriNetX Dataworks USA Network	NA	Yeh M.; Kuranz S.; Brzozowski K. et al	Changes in hemoglobin measures observed in PNH patients treated with both c5 inhibitors ravulizumab and eculizumab: Real-world evidence from a us-based EMR network	2021
	NA	Nagalla, S.; Hill, A.; Royston, M. et al	Ravulizumab and eculizumab reduce transfusions in adult patients with paroxysmal nocturnal hemoglobinuria: Evidence from three real-world databases: Trinetx us emr, trinetx us claims and komodo health	2021
UK National PNH Service	NA	Haughton J.; Kelly R.J.; Richards S.J. et al	Improved outcomes of budd-chiari syndrome in paroxysmal nocturnal hemoglobinuria with eculizumab therapy	2012
	NA	McKinley C.E.; Richards S.J.; Munir T. et al	Extravascular hemolysis due to C3-loading in patients with PNH treated with eculizumab: Defining the clinical syndrome	2017
	NA	Griffin M.; Hillmen P.; Munir T. et al	Concurrent treatment of aplastic anemia (AA) with immunosuppressive therapy and paroxysmal nocturnal hemoglobinuria (PNH) with eculizumab	2016
	NA	Griffin M.; Kulasekararaj A.; Gandhi S. et al	Concurrent treatment of aplastic anaemia (AA)/ paroxysmal nocturnal haemoglobinuria (PNH) syndrome with immunosuppressive therapy and eculizumab: A UK experience	2017
	NA	Arnold L.M.; Brooksbank G.L.; Kelly R.J. et al	Continued benefit from prolonged treatment with eculizumab in 130 patients with PNH in the UK: Home delivery of eculizumab is safe, convenient and associated with very high levels of patient satisfaction	2011
	NA	Dingli D.; Matos J.E.; Lehrhaupt K. et al	The burden of illness in patients with paroxysmal nocturnal hemoglobinuria receiving treatment with the C5-inhibitors eculizumab or ravulizumab: results from a US patient survey	2022
	NA	Karadag, Fatma Keklik; Yenerel, Mustafa Nuri; Yilmaz, Mehmet et al	Evaluation of clinical characteristics of patients with paroxysmal nocturnal hemoglobinuria treated with eculizumab in Turkey: a multicenter retrospective analysis.	2021
	NA	Kulagin A.; Klimova O.; Dobronravov A. et al	Incidence, clinical characteristics and outcome of symptomatic thromboembolic events (TE) in 276 patients with paroxysmal nocturnal hemoglobinurea (PNH)	2016
	NA	Kulagin A.; Klimova O.; Rudakova T. et al	Benefits and limitations of long-term eculizumab treatment for paroxysmal nocturnal hemoglobinuria (PNH): Real-world data from large cohort study in Russia	2018
	NA	Schaap C.C.M.; Heubel-Moenen F.C.J.I.; Nur E. et al	Nationwide study of eculizumab in paroxysmal nocturnal hemoglobinuria: Evaluation of treatment indications and outcomes	2023

Trial name	Primary or linked records?	Authors	Title	Published year
**Alexion database**	NA	Socie, G; Caby-Tosi, M; Marantz, J. L. et al	Eculizumab in paroxysmal nocturnal haemoglobinuria and atypical haemolytic uraemic syndrome: 10-year pharmacovigilance analysis	2019
**Small observational studies (n≤100)**				
—	NA	Alexander W.; Kelly R.J.	American Society of Hematology, 52nd Annual Meeting and Exposition: Improved survival with eculizumab (Soliris) in paroxysmal nocturnal hemoglobinuria	2011
—	NA	Chatzileontiadou S.; Hatjiharissi E.; Angelopoulou M. et al	Thromboembolic events in patients with paroxysmal nocturnal hemoglobinuria (PNH): Real world data of a Greek nationwide multicenter retrospective study	2023
—	NA	De Latour R.P.; Fremeaux-Bacchi V.; Porcher R. et al	Assessing complement blockade in patients with paroxysmal nocturnal hemoglobinuria receiving eculizumab	2015
—	NA	Debureaux P.-E.; Cacace F.; Silva B.G.P. et al	Hematological Response to Eculizumab in Paroxysmal Nocturnal Hemoglobinuria: Application of a Novel Classification to Identify Unmet Clinical Needs and Future Clinical Goals	2019
—	NA	Devalet B.; Wannez A.; Bailly N. et al	Prospective and comparative study of paroxysmal nocturnal hemoglobinuria patients treated or not by eculizumab: Focus on platelet extracellular vesicles	2019
—	NA	Dezern A.E.; Dorr D.; Brodsky R.A.	Predictors of hemoglobin response to eculizumab therapy in paroxysmal nocturnal hemoglobinuria	2013
—	NA	Dingli D.; Messer J.; Mohd Sani J. et al	Characterizing blood transfusion burden in patients with paroxysmal nocturnal hemoglobinuria: results from a patient chart audit survey	2023
—	NA	Fishman J, Rich C, Wilson K et al	Understanding the Real-World Clinical Effectiveness of Pegcetacoplan for the Treatment of Paroxysmal Nocturnal Hemoglobinuria (PNH)	2023
—	NA	Fureder W.; Sperr W.R.; Heibl S. et al	Prognostic factors and follow-up parameters in patients with paroxysmal nocturnal hemoglobinuria (PNH): experience of the Austrian PNH network	2020
—	NA	Goren Sahin D.; Akay O.M.; Keklik M. et al	Clinical characteristics and therapeutic outcomes of paroxysmal nocturnal hemoglobinuria patients in Turkey: a multicenter experience	2021
—	NA	Hanes V.; Pan J.; Chow V.	Clinical Characteristics of Patients with Paroxysmal Nocturnal Hemoglobinuria (PNH): A Retrospective Chart Review Study	2019

Trial name	Primary or linked records?	Authors	Title	Published year
—	NA	Iori A.P.; Quattrocchi L.; De Propris S. et al	Incidence and outcome of severe complications in patients with paroxysmal nocturnal hemoglobinuria: A real life scenario from a single center 30 years experience	2019
—	NA	Iori A.P.; Torelli G.F.; La Rocca U. et al	Paroxysmal nocturnal hemoglobinuria: A long-term single center experience	2017
—	NA	Kang K.-W.; Moon H.; Lee B.-H. et al	Nationwide study of paroxysmal nocturnal hemoglobinuria in South Korea: paradox of eculizumab	2020
—	NA	Kelly, Richard J; Hill, Anita; Arnold, Louise M et al	Long-term treatment with eculizumab in paroxysmal nocturnal hemoglobinuria: sustained efficacy and improved survival.	2011
—	NA	Munoz-Linares, Cristina; Ojeda, Emilio; Fores, Rafael et al	Paroxysmal nocturnal hemoglobinuria: a single Spanish center's experience over the last 40 yr.	2014
—	NA	Nakayama H.; Usuki K.; Echizen H. et al	Eculizumab dosing intervals longer than 17 days may be associated with greater risk of breakthrough hemolysis in patients with paroxysmal nocturnal hemoglobinuria	2016
—	NA	Panse, Jens; Sicre de Fontbrune, Flore; Burmester, Pascale et al	The burden of illness of patients with paroxysmal nocturnal haemoglobinuria receiving C5 inhibitors in France, Germany and the United Kingdom: Patient-reported insights on symptoms and quality of life.	2022
—	NA	Plessier A.; Esposito-Farese M.; Baiges A. et al	Paroxysmal nocturnal hemoglobinuria and vascular liver disease: Eculizumab therapy decreases mortality and thrombotic complications	2022
—	NA	Plessier A.; Rene B.; Baiges A. et al	Paroxysmal nocturnal hemoglobinuria and Budd Chiari syndrome: Impact of Eculizumab therapy on survival and liver outcome in 54 patients: A multicentric valdig study	2019
—	NA	Roth A.; Hock C.; Konik A. et al	Chronic treatment of paroxysmal nocturnal hemoglobinuria patients with eculizumab: Safety, efficacy, and unexpected laboratory phenomena	2011
—	NA	Sahin F.; Keklik Karadag F.; Saydam G.	The evaluation of paroxismal nocturnal hemoglobinuria patients who underwent eculizumab therapy	2019
—	NA	Schrezenmeier H.; Schubert J.; Luzzatto L. et al	Effects of eculizumab therapy in patients with paroxysmal nocturnal hemoglobinuria (PNH) receiving concurrent immunosuppressive therapy for bone marrow insufficiency	2009
—	NA	Shammo J.; Gajra A.; Patel Y. et al	Low Rate of Clinically Evident Extravascular Hemolysis in Patients with Paroxysmal Nocturnal Hemoglobinuria Treated with a Complement C5 Inhibitor: Results from a Large, Multicenter, US Real-World Study	2022

Trial name	Primary or linked records?	Authors	Title	Published year
—	NA	Sicre de Fontbrune, Flore; Burmester, Pascale; Piggin, Maria et al	The burden of illness of patients with paroxysmal nocturnal haemoglobinuria receiving C5 inhibitors: clinical outcomes and medical encounters from the patient perspective.	2022
—	NA	Subias Hidalgo M.; Martin Merinero H.; Lopez A et al	Extravascular hemolysis and complement consumption in Paroxysmal Nocturnal Hemoglobinuria patients undergoing eculizumab treatment	2017
—	NA	Versmold K.; Alashkar F.; Raiser C. et al	Clinical profile and long-term outcomes of patients with paroxysmal nocturnal hemoglobinuria treated with eculizumab in a real-world setting: High frequency of anemia despite decreased intravascular hemolysis	2021
—	NA	Versmold K.; Alashkar F.; Raiser C. et al	Long-term outcomes of patients with paroxysmal nocturnal hemoglobinuria treated with eculizumab in a real-world setting	2023
—	NA	Weitz I.C.; Razavi P.; Rochanda L. et al	Eculizumab therapy results in rapid and sustained decreases in markers of thrombin generation and inflammation in patients with PNH independent of its effects on hemolysis and microparticle formation	2012

†Study 307 OLE is included in the complement-inhibitor naïve and complement-inhibitor experienced sections, as it provides data for both populations.
Abbreviations: NA, not applicable.

Outcomes in the CI-naïve population

Efficacy outcomes reported in clinical trials

Supplementary Table S2: Efficacy outcomes reported in clinical trials evaluating patients with CI-naïve PNH

Clinical trial name or acronym<br>Linked record[†]	Intervention	N	Timepoint	Mean CFB Hb, g/dL	Hb stabilization or hematological response, n (%)	Transfusion avoidance, n (%)	No. of PRBC units	Mean CFB LDH, g/L	LDH normalization, n (%)	Mean CFB ARC, 10^9/L
**Eculizumab clinical trials**										
**TRIUMPH**<br>Hillmen 2006<br>Schubert 2008	ECU	43	Week 26	NR	21 (49)[‡]	22 (51)[¶]	3 [SD: 0.67][‡]	NR	NR	NR
	PBO	44	Week 26	NR	0 (0) [‡]	0 (0)[¶]	11 [SD: 0.83][‡]	NR	NR	NR

Clinical trial name or acronym<br>Linked record[†]	Intervention	N	Timepoint	Mean CFB Hb, g/dL	Hb stabilization or hematological response, n (%)	Transfusion avoidance, n (%)	No. of PRBC units	Mean CFB LDH, g/L	LDH normalization, n (%)	Mean CFB ARC, 10^9/L
**SHEPHERD** Brodsky 2008	ECU	97	Week 52	NR	NR	49 (51)	5.9 [SE: 1.06]	NR	97 (100)[‡]	NR
**Eculizumab extension study** (including patients from TRIUMPH, SHEPHERD, and Hillmen 2004)<br>Hillmen 2011<br>Hillmen 2013	ECU	195	Week 2	0.55 [SD: 0.096]	NR	NR	NR	NR	NR	NR
		195	Month 1	0.95	NR	NR	NR	NR	NR	NR
		195	Month 24	1.04 [SD: 0.159]	NR	NR	4.6 [SD: 0.61]	NR	NR	NR
		195	Month 36	0.95 [0.22]	NR	NR	2.4 [SD: 0.3]	NR	NR	NR
**Ravulizumab clinical trials**										
**Study 301**<br>Lee 2019<br>Schrezenmeier 2020<br>Kulasekararaj 2022<br>Kulasekararaj 2023[§]	RAV	125	Week 26	NR	85 (68.0)[¶]	92 (73.6)	4.8 [SD: 5.1][¶]	NR	NR (53.6)[‡]	NR
	ECU	121	Week 26	NR	78 (64.5)[¶]	80 (66.1)	5.6 [SD: 5.9][¶]	NR	NR (49.4)[‡]	NR
	RAV to RAV	124	Week 27 to Week 52	NR	91 (73.4)	95 (76.6)	NR	NR	54 (43.5)	NR
	ECU to RAV	119	Week 27 to Week 52	NR	78 (65.5)	80 (67.2)	NR	NR	48 (40.3)	NR
	RAV (including ECU to RAV and RAV to RAV)	NR	Two years	NR	NR (69.1)	NR (81.9)	NR	NR	108 (48.2)	NR
	RAV (including ECU to RAV and RAV to RAV)	53	Day 2045	NR	NR	NR (75.7)	110	NR	42 (79.3)	NR

Clinical trial name or acronym<br>Linked record[†]	Intervention	N	Timepoint	Mean CFB Hb, g/dL	Hb stabilization or hematological response, n (%)	Transfusion avoidance, n (%)	No. of PRBC units	Mean CFB LDH, g/L	LDH normalization, n (%)	Mean CFB ARC, 10^9/L
**Pegcetacoplan clinical trials**										
**PRINCE**<br>Wong 2023[§]<br>Wong 2022<br>Gomez-Almaguer 2022<br>NCT record	PEG	35	Week 26	2.9[¶,††]	30 (85.7)[‡]	32 (91.4)[¶]	NR	–1870.5[‡,††]	23 (65.7)[¶]	–123.3[¶]
	SOC	18		0.3[¶,††]	0 (0.0)[‡]	1 (5.6)[¶]	NR	–400.1[‡,††]	0 (0)[¶]	–19.4[¶]
**Study 307 OLE[¶¶]**<br>Patriquin 2024	PEG	50	Week 48	NR	8 (28.6)	45 (90.0)	NR	NR	17 (63.0)	NR
**Iptacopan clinical trials**										
**APPOINT-PNH**<br>De Latour 2024[§]	IPTA	40	Between Day 14 and Day 168	NR	NR	40 (97.6)[¶]	1.7 [SD: 0.45](8)	NR	NR	NR
		40	Between Day 126 and Day 168[‡‡]	4.3 (95%CI 3.9, 4.7) [¶]	31 (92.2)[‡]	NR	NR	NR	NR	–82.48 (95%CI –89.33, –75.62)[¶]
**Crovalimab clinical trials**										
**COMMODORE 2**<br>Roth 2024[§]<br>Lundberg 2023[§]	CROVA	135	Week 5	NR	NR	NR	NR	NR	NR (81)	NR
	ECU	69	Week 5	NR	NR	NR	NR	NR	NR (84)	NR
	CROVA	135	Week 25	1.2 [SD: 2.0]	85 (63.4)[¶]	88 (65.7)[‡]	2.33 [SD: 6.02]	NR	NR (79.3)[‡]	NR
	ECU	69	Week 25	0.8 [SD: 1.9]	42 (60.9)[¶]	47 (68.1)[‡]	2.20 [SD: 4.83]	NR	NR (79.0)[‡]	NR
**COMMODORE 3**<br>Liu 2023[§]	CROVA	51	Primary cut-off: February 10th 2022	NR	26 (51.0)[¶]	26 (51.0)[‡]	4.6 [SD: 6.7]	NR	NR (78.7)[‡]	NR

Clinical trial name or acronym<br>Linked record†	Intervention	N	Timepoint	Mean CFB Hb, g/dL	Hb stabilization or hematological response, n (%)	Transfusion avoidance, n (%)	No. of PRBC units	Mean CFB LDH, g/L	LDH normalization, n (%)	Mean CFB ARC, 10^9/L
**Eculizumab biosimilar clinical trials**										
**NCT04058158<br>Jang 2023**	SB12 (ECU biosimilar) to ECU	24	Week 26	NR	NR	NR	1.1 [SD: 3.72] ¶	NR	NR	NR
	ECU to SB12 (ECU biosimilar)	25		NR	NR	NR	0.9 [SD: 2.06] ¶	NR	NR	NR
	SB12 (ECU biosimilar) to ECU	24	Week 52	NR	NR	NR	1.1 [SD: 4.05] ¶	NR	NR	NR
	ECU to SB12 (ECU biosimilar)	25		NR	NR	NR	1.0 [SD: 2.61] ¶	NR	NR	NR

†Efficacy outcomes have been sourced from all referenced records per clinical trial in this table. If different timepoints are reported in separate records, it has been indicated which record reports each timepoint; ‡Primary endpoint; ¶Secondary endpoint; §Records identified in the September 2024 SLR update; ††least squares mean; ‡‡The two primary end points were an increase in Hb level of at least 2 g per deciliter from baseline and a Hb level of at least 12 g per deciliter (both as measured on at least three of four assessments between days 126 and 168 [day 1 was the day of the first dose], a time frame chosen to allow investigation of a durable hematologic response and to show the actual treatment effect of iptacopan or anti-C5), each without red-cell transfusions between days 14 and 168 or without meeting the protocol-specified criteria for red-cell transfusion; ¶¶Only data from patients who were enrolled in the Phase 3 PRINCE trial and the 307 OLE study met the PICOS eligibility criteria.

Abbreviations: ARC, absolute reticulocyte count; BTH, breakthrough hemolysis; CFB, change from baseline; CI-naïve, complement inhibitor-naïve; CROVA, crovalimab; DANI, danicopan; ECU, eculizumab; Hb, hemoglobin; IPTA, iptacopan; LDH, lactate dehydrogenase; NR, not reported; PEG, pegcetacoplan; PICOS, Population, Intervention, Comparator(s), Outcome(s), Study design; PRBC, packed red blood cell; RAV, ravulizumab; SD, standard deviation; SE, standard error; SLR, systematic literature review; SoC; standard-of-care.

Supplementary Table S3: FACIT-Fatigue reported in clinical trials evaluating patients with CI-naïve PNH

Clinical trial name or acronym Linked records[†]	N	Intervention	Timepoint	Mean FACIT-Fatigue score at baseline	Mean CFB FACIT-Fatigue score
**Eculizumab clinical trials**					
**TRIUMPH** Hillmen 2006	43	ECU	Week 26[‡]	NR	6.4 [SE: 1.19]
	44	PBO	Week 26[‡]	NR	–4.0 [SE: 1.71]
**SHEPHERD** Brodsky 2008	97	ECU	Week 52[‡]	NR	12.2 [SE: 1.09]
**Ravulizumab clinical trials**					
**Study 301** Lee 2019 Schrezenmeier 2020	125	RAV	Week 26[‡](4)	NR	7.07 (95% CI: 5.55, 8.60)
	121	ECU	Week 26[‡](4)	NR	6.40 (95% CI: 4.85, 7.96)
	125	RAV to RAV	Week 52(5)	NR	7.5 (SD/SE NR)
	121	ECU to RAV	Week 52(5)	NR	6.4 (SD/SE NR)
**Pegcetacoplan clinical trials**					
**PRINCE** Wong 2023	35	PEG	Week 26[‡]	36.30 [SD: 10.66]	7.8 [SE: 1.2]
	18	SoC	Week 26[‡]	37.10 [SD:9.32]	3.3 [SE: 2.1]
**Iptacopan clinical trials**					
**APPOINT-PNH** De Latour 2024	39	IPTA	Day 126 to Day 168[‡]	32.78 [SD: 10.17]	10.75 (95% CI: 8.66, 12.84)
**Crovalimab clinical trials**					
**COMMODORE 2** Panse 2023	128	CROVA	Week 25	NR	7.79 [SE: 0.661]
	66	ECU	Week 25	NR	5.15 [SE: 0.88]
**COMMODORE 3** Liu 2023	51	CROVA	Week 17[‡]	31.8 (95% CI: 29.3, 34.3)	8.8 (95% CI: 6.0, 11.6)

†HRQoL outcomes have been sourced from all referenced records per clinical trial in this table. If different timepoints are reported in separate records, it has been indicated which record reports each timepoint; ‡Secondary or exploratory endpoint.
Abbreviations: CFB, change from baseline; CI, confidence interval; CROVA, crovalimab; ECU, eculizumab; FACIT-Fatigue, Functional Assessment of Chronic Illness Therapy – Fatigue; HRQoL, health-related quality of life; IPTA, iptacopan; PEG, pegcetacoplan; PNH, paroxysmal nocturnal hemoglobinuria; NR, not reported; PBO, placebo; QoL, quality of life; RAV, ravulizumab; SD, standard deviation; SE, standard error; SoC, standard-ofcare.

Breakthrough hemolysis and safety outcomes reported in clinical trials

Supplementary Table S4: BTH and safety outcomes reported in clinical trials evaluating patients with CI-naïve PNH

Clinical trial name or acronym<br>Linked records[†]	N	Intervention	Timepoint	Any-Grade all-cause AEs, n (%)	Any-Grade treatment-related AEs, n (%)	SAEs, n (%)	MAVEs, n (%)	BTH, n (%)	Hemolysis AE, n (%)	Discontinuations, n (%)		
										Total	Due to any AE	Due to treatment-related AEs
**Eculizumab clinical trials**												
**TRIUMPH**<br>Hillmen 2006	43	ECU	Week 26	NR	NR	4 (9)	0 (0)	NR	NR	2 (4.7)	0 (0)	0 (0)
	44	PBO	Week 26	NR	NR	9 (20)	1 (2.3)	NR	NR	20 (22.7)	0 (0)	0 (0)
**SHEPHERD**<br>Brodsky 2008	97	ECU	Week 52	NR	2 (2.1)	44 (45.4)[‡]	2 (2.1)	8 (8.2)	8 (8.2)	1 (1.0)	1 (1.0)	0 (0.0)
**Eculizumab extension study** (including patients from Hillmen 2004, SHEPHERD, and TRIUMPH)<br>Hillmen 2013	195	ECU	Month 36	NR	NR	NR	NR	NR	NR	NR	NR	NR
	195	ECU	Month 66	194 (99.5)	NR	75 (38.5)	7 (3.6)	NR	NR	19 (9.7)	9 (4.6)	NR
**Ravulizumab clinical trials**												
**Study 301**<br>Lee 2019<br>Schrezenmeier 2020<br>Kulasekararaj 2023	125	RAV	Week 26	110 (88.0)	NR	11 (8.8)	2 (1.6)[¶]	5 (4.0)	5 (4.0)	0 (0)	0 (0)	NR
	121	ECU	Week 26	105 (86.8)	NR	9 (7.4)	1 (0.8)[¶]	13 (10.7)	13 (10.7)	2 (1.7)	1 (0.8)	NR
	124	RAV to RAV	Week 27 to Week 52	79 (63.7)	NR	9 (7.3)	0 (0)[¶]	NR	4 (3.2)	NR	0 (0)	NR

Clinical trial name or acronym<br>Linked records[†]	N	Intervention	Timepoint	Any-Grade all-cause AEs, n (%)	Any-Grade treatment-related AEs, n (%)	SAEs, n (%)	MAVEs, n (%)	BTH, n (%)	Hemolysis AE, n (%)	Discontinuations, n (%)		
										Total	Due to any AE	Due to treatment-related AEs
	119	ECU to RAV	Week 27 to Week 52	89 (74.8)	NR	7 (5.9)	1 (0.8)[¶]	NR	2 (1.6)	NR	1 (0.8)	NR
	53	RAV	Day 2,045	NR	NR	NR	9 (3.7)	NR	NR	33 (13.4)	3 (5.6)	NR
**Pegcetacoplan clinical trials**												
**PRINCE**<br>Wong 2023[§]	25	PEG	Week 26	33 (71.7)	13 (28.3)	4 (8.7)	0 (0)	NR	NR	2 (NR)	0 (0)	NR
	18	SoC		12 (66.7)	NR	3 (16.7)	0 (0)	NR	NR	12 (NR)	0 (0)	NR
**Study 307 OLE**[††]<br>Patriquin 2024	PEG	50	Week 48	23 (46.0)	5 (10.0)	6 (12.0)	NR	NR	6 (12.0)	NR	1 (2)	NR
**Iptacopan clinical trials**												
**APPOINT-PNH**<br>De Latour 2024	40	IPTA	Week 24	37 (92)	14 (35.0)	4 (10)	0 (0)	0 (0)	0 (0)	0 (0)	0 (0)	0 (0)
**Crovalimab clinical trials**												
**COMMODORE 2**<br>Roth 2024[§]	135	CROVA	Week 25	105 (77.8)	45 (33.3)	14 (10.4)	1 (0.74)	14 (10.4)	NR	NR	1 (0.7)	NR
	69	ECU	Week 25	55 (79.7)	24 (34.8)	9 (13)	1 (1.45)	10 (14.5)	NR	NR	1 (1.4)	NR
**COMMODORE 3**<br>Liu 2023[§]	51	CROVA	Primary cut-off: February 10th 2022	50 (98)	39 (76)	4 (8)	0 (0)	2 (3.9)	NR	1 (2)	0 (0)	NR

Clinical trial name or acronym<br>Linked records†	N	Intervention	Timepoint	Any-Grade all-cause AEs, n (%)	Any-Grade treatment-related AEs, n (%)	SAEs, n (%)	MAVEs, n (%)	BTH, n (%)	Hemolysis AE, n (%)	Discontinuations, n (%)		
										Total	Due to any AE	Due to treatment-related AEs
**Eculizumab biosimilar clinical trials**												
**NCT04058158**<br>Jang 2023	47	ECU	Week 52	32 (68)	8 (17)	2 (4)	1 (2)	1 (2)	0 (0)	2 (4)	1 (2)	0 (0)
	47	SB12	Week 52	34 (72)	3 (6)	3 (6)	0 (0)	8 (17)	1 (2)	1 (2)	0 (0)	0 (0)

†Safety outcomes have been sourced from all referenced records per clinical trial in this table. If different timepoints are reported in separate records, it has been indicated which record reports each timepoint; ‡Number of SAEs; ¶Secondary endpoint; §Records identified in the September 2024 SLR update; ††Only data from patients who were enrolled in the Phase 3 PRINCE trial and the 307 OLE study met the PICOS eligibility criteria.

Abbreviations: AE, adverse event; BTH, breakthrough hemolysis; CI-naïve, complement inhibitor-naïve; CROVA, crovalimab; DANI, danicopan; ECU, eculizumab; IPTA, iptacopan; MAVE, major adverse vascular event; NR, not reported; PBO, placebo; PEG, pegcetacoplan; PICOS, Population, Intervention, Comparator(s), Outcome(s), Study design; PNH, paroxysmal nocturnal hemoglobinuria; RAV, ravulizumab; SAE, serious adverse event; SLR, systematic literature review update; SoC, standard-of-care.

Outcomes in the CI-experienced population

Efficacy outcomes reported in clinical trials

Supplementary Table S5: Efficacy outcomes reported in clinical trials evaluating patients with CI-experienced PNH and residual anemia

Clinical trial name or acronym<br>Linked records[†]	Intervention	N	Timepoint	Mean CFB Hb level, g/dL	Hb stabilization or hematological response, n (%)	Transfusion avoidance, n (%)	No. of PRBC units transfused	Mean CFB LDH level, g/dL	LDH normalization, n (%)	Mean CFB ARC, 10^9/L
**Pegcetacoplan clinical trials**										
**PEGASUS**<br>Hillmen 2021<br>De Latour 2022<br>Mulherin 2022<br>NCT record	PEG	41	Week 16	2.37 [SE: 0.36][‡]	14 (34.1)	35 (85)[¶]	NR	–15.0 [SE: 42.7][¶]	29 (70.7)	–136 [SE: 6.5][¶]
	ECU	39		–1.47 [SE: 0.67][‡]	0 (0)	6 (15)[¶]	NR	–10.0 [SE: 71.0][¶]	6 (15.4)	28 [SE: 11.9][¶]
	PEG to PEG	41	Week 48	NR	10 (24.4)	30 (73)	NR	2.47 [SD: 1.72]	23 (56.1)	NR
	ECU to PEG	39		NR	12 (30.8)	28 (72)	NR	2.93 [SD: 2.09]	20 (51.3)	NR
	PEG to PEG	33	Week 17 to Week 48	–0.16 [SD: 1.154]	NR	NR	NR	8.03 [SD: 129.285]	NR	–6.50 [SD: 26.471]
	ECU to PEG	29		2.89 [SD: 2.078]	NR	NR	NR	–46.84 [SD: 292.607]	NR	–121.15 [SD: 70.969]
**Study 307 OLE**[§]<br>Patriquin 2024	PEG	64	Week 48	NR	18 (31.6)	49 (76.6)	NR	NR	40 (70.2)	NR
**Iptacopan clinical trials**										
**APPLY-PNH**<br>De Latour 2024[††]	IPTA	62	Day 126 to Day 168[‡‡]	3.6 (95% CI: 3.3, 3.9)[¶]	NR	NR	NR	NR	NR	–115.8[¶¶]
	C5i	30		–0.06 (95%CI –0.5, 0.3)[¶]	NR	NR	NR	NR	NR	0.3[¶¶]
	IPTA	62	Day 14 to Day 168	NR	NR	59 (94.8[§§])	NR	NR	NR	NR

Clinical trial name or acronym<br>Linked records†	Intervention	N	Timepoint	Mean CFB Hb level, g/dL	Hb stabilization or hematological response, n (%)	Transfusion avoidance, n (%)	No. of PRBC units transfused	Mean CFB LDH level, g/dL	LDH normalization, n (%)	Mean CFB ARC, 10^9/L
	C5i	35		NR	NR	14 (25.9§§)	NR	NR	NR	NR
**Danicopan clinical trials**										
**ALPHA**<br>Lee 2023††<br>Kulasekararaj 2023‡‡	DANI + ECU/RAV	49	Week 12	2.94 (95% CI: 2.52, 3.36)‡	25 (60)§	35 (83)¶	NR	–23.49 (95% CI: –40.08, –6.90)¶	NR	–83.8 (95% CI: –101.6, –65.9)¶
	PBO + ECU/RAV	24	Week 12	0.50 (95% CI: –0.13, 1.12)‡	0 (0)§	8 (38)¶	NR	–2.92 (95% CI: –26.78, 20.93)¶	NR	3.5 (95% CI: –21.9, 28.8)¶
	DANI + ECU/RAV	57	Week 24	3.17 [SD: 0.3]	19 (46.3)§	32 (78)¶	NR	NR	NR	–80.2 [SE: 8.75]
	PBO + ECU/RAV	29	Week 24	2.26 [SD: 0.34]	7 (35)§	18 (90)¶	NR	NR	NR	–65.2 [SE: 12.74]

†Efficacy outcomes have been sourced from all referenced records per clinical trial in this table. If different timepoints are reported in separate records, it has been indicated which record reports each timepoint; ‡Primary endpoint; ¶Secondary endpoint; §Only data from patients who were enrolled in the Phase 3 PEGASUS trial and the 307 OLE study met the PICOS eligibility criteria; ††Records identified in the September 2024 SLR update; ‡‡ An increase in Hb of at least 2 g per deciliter from baseline without red-cell transfusion was the primary end point, and a Hb level of at least 12 g per deciliter without red-cell transfusion was a secondary end point (both as measured on at least three of four assessments between days 126 and 168 [day 1 was the day of the first dose], a time frame chosen to allow investigation of a durable hematologic response and to show the actual treatment effect of iptacopan or anti-C5), each without red-cell transfusions between days 14 and 168 or without meeting the protocol-specified criteria for red-cell transfusion; ¶¶Hb and LDH levels at follow-up were the only outcomes reported; §§Reported as marginal proportion calculated using logistic regression model with common intercept and randomization strata, sex, indicator variable of age ≥45 years, indicator variable of baseline hemoglobin ≥9 g/dL as factors.

Abbreviations: ARC, absolute reticulocyte count; C5i, C5 inhibitors; CFB, change from baseline; CI, confidence interval; CI-experienced, complement inhibitor-experienced; DANI, danicopan; ECU, eculizumab; Hb, hemoglobin; IPTA, iptacopan; LDH, lactate dehydrogenase; NR, not reported; PBO, placebo; PEG, pegcetacoplan; PICOS, Population, Intervention, Comparator(s), Outcome(s), Study design; PRBC, packed red blood cell; RAV, ravulizumab; SD, standard deviation; SE, standard error; SLR, systematic literature review.

Supplementary Table S6: FACIT-Fatigue reported in clinical trials evaluating patients with CI-experienced PNH and residual anemia

Clinical trial name or acronym<br>Linked records[†]	N	Intervention	Timepoint	Mean FACIT-Fatigue score at baseline	Mean CFB FACIT-Fatigue score
**Pegcetacoplan clinical trials**					
**PEGASUS**<br>Hillmen 2021<br>De Latour 2022	41	PEG	Week 16[‡](11)	32.2 [SD: 11.4]	9.2 [SE: 1.6]
	39	ECU	Week 16[‡](11)	31.6 [SD: 12.5]	–2.7 [SE: 2.8]
	41	PEG	Week 48(12)	32.2 [SD: 11.4]	10.14 [SD: 9.06]
	39	ECU	Week 48(12)	31.6 [SD: 12.5]	9.62 [SD: 10.34]
**Iptacopan clinical trials**					
**APPLY-PNH**<br>De Latour 2024	62	IPTA	Day 168[‡]	34.7 [SD: 9.82]	8.6 (95% CI: 6.7, 10.5)
	35	C5i	Day 168[‡]	30.8 [SD: 10.17]	0.3 (95% CI: –2.2, 2.8)
**Danicopan clinical trials**					
**ALPHA**<br>Lee 2023<br>Piatek 2023	49	DANI + ECU/RAV	Week 12[‡](15)	34.19 [SD: 11.01]	7.97 (95% CI: 5.72, 10.23)
	24	PBO + ECU/RAV	Week 12[‡](15)	33.61 [SD: 10.74]	1.85 (95% CI: –1.31, 5.02)
	40	DANI + ECU/RAV	Week 24(17)	40.32 [SD: NR]	6.12 [SD: 1.34]
	20	PBO + ECU/RAV	Week 24(17)	40.55 [SD: NR]	6.44 [SD: 2.47]

†HRQoL outcomes have been sourced from all referenced records per clinical trial in this table. If different timepoints are reported in separate records, it has been indicated which record reports each timepoint; ‡Secondary or exploratory endpoint.

Abbreviations: C5i, C5 inhibitors; CFB, change from baseline; CI, confidence interval; DANI, danicopan; ECU, eculizumab; FACIT-Fatigue, Functional Assessment of Chronic Illness Therapy – Fatigue; HRQoL, health related quality of life; IPTA, iptacopan; NR, not reported; PBO, placebo; PNH, paroxysmal nocturnal hemoglobinuria; QoL, quality of life; RAV, ravulizumab; SD, standard deviation; SE, standard error.

Breakthrough hemolysis and safety outcomes reported in clinical trials

Supplementary Table S7: BTH and safety outcomes reported in clinical trials evaluating patients with CI-experienced PNH and residual anemia

Clinical trial name or acronym Linked records†	Intervention	N	Timepoint	Any-Grade all-cause AEs, n (%)	Any-Grade treatment-related AEs, n (%)	SAEs, n (%)	MAVEs, n (%)	BTH, n (%)	Hemolysis AE, n (%)	Discontinuations, n (%)		
										Total	All-cause AEs	Treatment-related AEs
**Pegcetacoplan clinical trials**												
**PEGASUS** Hillmen 2021 De Latour 2022 Mulherin 2024‡	PEG	41	Week 16	36 (88)	NR	7 (17)	0 (0)	4 (10)	4 (10)	3 (7)	3 (7)	NR
	ECU	39		34 (87)	NR	6 (15)	0 (0)	9 (23)	9 (23)	0 (0)	0 (0)	NR
	PEG to PEG	38	Week 1 to Week 48	33 (87)	NR	8 (21)	1 (3)	NR	7 (18)	NR	NR	0 (0)
	ECU to PEG	39		37 (95)	NR	10 (26)	1 (3)	NR	8 (21)	NR	NR	0 (0)
	PEG to PEG and ECU to PEG	77	Week 48	71 (92.2)	34 (44.2)	18 (23.4)	NR	NR	NR	NR	9 (11.7)	4 (5.2)
**Study 307 OLE¶** Patriquin 2024	PEG	64	Week 48	60 (93.8)	13 (20.3)	16 (25)	NR	NR	14 (21.9)	NR	2 (3.1)	NR
**Iptacopan clinical trials**												
**APPLY-PNH** De Latour 2024‡	IPTA	62	Week 24	51 (82)	16 (25.8)(18)	6 (10)	1 (1.6)	2 (3)	NR	NR	0 (0)	0 (0)
	C5i	35		28 (80)	3 (8.6)(18)	5 (14)	0 (0)	6 (17)	NR	NR	0 (0)	0 (0)
**Danicopan clinical trials**												
**ALPHA** Lee 2023‡	DANI + ECU/RAV	49	Week 12	35 (71)	9 (18)	2 (4)	NR	NR	2 (4)	2 (NR)	2 (NR)	2 (NR)
	PBO + ECU/RAV	24	Week 12	15 (63)	7 (29)	1 (4)	NR	NR	0 (0)	1 (NR)	1 (NR)	1 (NR)

†Safety outcomes have been sourced from all referenced records per clinical trial in this table. If different timepoints are reported in separate records, it has been indicated which record reports each timepoint. ‡Records identified in the September 2024 SLR update. ¶Only data from patients who were enrolled in the Phase 3 PEGASUS trial and the 307 OLE study met the PICOS eligibility criteria.

Abbreviations: AE, adverse event; BTH, breakthrough hemolysis; C5i, C5 inhibitors; CI-experienced, complement inhibitor-experienced; DANI, danicopan; ECU, eculizumab; IPTA, iptacopan; MAVE, major adverse vascular event; NR, not reported; PBO, placebo; PEG, pegcetacoplan; PNH, paroxysmal nocturnal hemoglobinuria; RAV, ravulizumab; SAE, serious adverse event; SLR, systematic literature review.

Definitions of hemoglobin-related and hemolysis-related efficacy outcomes

Supplementary Table S8: Hemoglobin-related and hemolysis-related definitions provided in trials reporting these outcomes

Clinical trial name	Hb outcome	Definition of Hb outcome
**Clinical trials evaluating patients with CI-naïve PNH**		
**TRIUMPH**<br>Hillmen 2006	Hb stabilization	Hb stabilization was reported as a primary endpoint<br>A Hb value that was maintained above the level in which the qualifying transfusion was administered, in the absence of transfusions
	Hemolysis	Hemolysis was reported as a secondary endpoint<br>As measured by LDH AUC
**SHEPHERD**<br>Brodsky 2008	Hemolysis	Hemolysis was reported as a primary endpoint<br>As assessed by LDH AUC
**Study 301**<br>Lee 2019	Hemolysis	Hemolysis was reported as a primary endpoint<br>As measured by LDH normalization [ULN 246 U/L]
	Hb stabilization	Hb stabilization was reported as a secondary endpoint<br>Avoidance of a ≥2 g/dl decrease in Hb level from baseline in the absence of transfusion
**PRINCE**<br>Wong 2023	Hb stabilization	Hb stabilization was reported as a primary endpoint<br>Avoidance of a >1 g/dl decrease in Hb levels from baseline
	Hb response	Hb response was reported as a secondary endpoint<br>A Hb increase of ≥1 g/dl from baseline
**APPOINT-PNH**<br>De Latour 2024	Hematological response	Hematological response was reported as a primary endpoint<br>Proportion of patients achieving a sustained increase in Hb levels of ≥2 g/dl in the absence of RBC transfusion
**COMMODORE 2**<br>Roth 2024	Hemolysis control	Hemolysis control was reported as a primary endpoint<br>LDH ≤1.5 x ULN
	Hb stabilization	Hb stabilization was reported as a secondary endpoint<br>Avoidance of a ≥2 g/dl decrease in Hb from baseline in the absence of transfusion
**COMMODORE 3**<br>Liu 2023	Hemolysis control	Hemolysis control was reported as a primary endpoint<br>LDH ≤1.5 x ULN
	Hb stabilization	Hb stabilization was reported as a secondary endpoint<br>Avoidance of a ≥2 g/dl decrease in Hb from baseline in the absence of transfusion
**NCT04058158**<br>Jang 2023	Reduction of hemolysis	Reduction of hemolysis was reported as a primary endpoint<br>Assessed by LDH levels and time-adjusted area under the effect curve of LDH from Weeks 14 to 26 and 40 to 52
**Clinical trials evaluating patients with CI-experienced PNH**		
**APPLY-PNH**<br>De Latour 2024	Hematological response	Hematological response was reported as a primary outcome<br>% of patients with a ≥2 g/dL Hb increase from baseline and the % of patients with Hb ≥12 g/dL, each in the absence of RBC transfusion

Abbreviations: CI, complement inhibitor; Hb, hemoglobin; LDH, lactate dehydrogenase; PNH, paroxysmal nocturnal hemoglobinuria; RBC, red blood cell; ULN, Upper Limit of Normal.

Lactate dehydrogenase (LDH) normalization definition

Supplementary Table S9: LDH normalization definitions provided in trials reporting this outcome

Clinical trial name	Definition of LDH
**Clinical trials evaluating patients with CI-naïve PNH**	
**Study 301**<br>Lee 2019	LDH normalization formed the definition of the primary endpoint of hemolysis<br>Upper limit normal 246 U/L
**APPOINT-PNH**<br>De Latour 2024	LDH normalization was reported as an efficacy endpoint<br>>1.5 times the upper limit of the normal range
**Clinical trials evaluating patients with CI-experienced PNH**	
**PEGASUS**<br>Hillmen 2021	LDH normalization was reported as a secondary endpoint<br>Upper limit normal 246 U/L

Abbreviations: CI, complement inhibitor; LDH, lactate dehydrogenase; PNH, paroxysmal nocturnal hemoglobinuria.

Breakthrough hemolysis (BTH) definition

Supplementary Table S10: BTH definitions provided in trials reporting this outcome

Clinical trial name	Definition of BTH
**Clinical trials evaluating patients with CI-naïve PNH**	
**SHEPHERD**<br>Brodsky 2008	BTH was reported as an efficacy endpoint<br>Definition: A return of terminal complement activity and hemolysis
**Study 301**<br>Lee 2019	BTH was reported as a key secondary endpoint<br>Definition: ≥1 new or worsening sign or symptom of intravascular hemolysis (fatigue, hemoglobinuria, abdominal pain, dyspnea, anemia [hemoglobin ,10 g/dL], MAVEs including thrombosis, dysphagia, or erectile dysfunction) in the presence of LDH ≥2X ULN after prior reduction of LDH to <1.5X ULN on treatment
**APPOINT-PNH**<br>De Latour 2024	BTH was reported as a secondary endpoint<br>Definition: Meeting one of the two clinical criteria (decrease in hemoglobin level ≥2 g per deciliter or PNH symptoms of gross hemoglobinuria, hemolytic crisis, dysphagia, or any other clinically significant sign or symptom associated with PNH) in addition to elevated LDH level (>1.5 times the upper limit of the normal range)
**COMMODORE 2**<br>Roth 2024	BTH was reported as a secondary efficacy endpoint<br>Definition: One or more new or worsening symptoms or signs of intravascular hemolysis in the presence of elevated LDH ⩾2 x ULN after prior reduction of LDH to ⩽1.5 x ULN on treatment
**COMMODORE 3**<br>Liu 2023	BTH was reported as an efficacy endpoint<br>Definition: One or more new or worsening symptoms or signs of intravascular hemolysis in the presence of elevated LDH ⩾2 x ULN after prior reduction of LDH to ⩽1.5 x ULN on treatment
**NCT04058158**<br>Jang 2023	BTH was reported as an efficacy endpoint (no definition)
**Clinical trials evaluating patients with CI-experienced PNH**	
**PEGASUS**<br>Hillmen 2021	BTH was reported as an adverse event (patients with BTH had an elevation of LDH level to more than 3 times the upper limit of the normal range)
**APPLY-PNH**<br>De Latour 2024	BTH was reported as a secondary endpoint<br>Definition: Meeting one of the two clinical criteria (decrease in hemoglobin level ≥2 g per deciliter or PNH symptoms of gross hemoglobinuria, hemolytic crisis, dysphagia, or any other

Clinical trial name	Definition of BTH
	clinically significant sign or symptom associated with PNH) in addition to elevated LDH level (>1.5 times the upper limit of the normal range)

Abbreviations: BTH, breakthrough hemolysis; CI, complement inhibitor; LDH, lactate dehydrogenase; MAVE, major adverse vascular event; PNH, paroxysmal nocturnal hemoglobinuria; ULN, upper limit of normal.

Risk of bias assessment

Supplementary Table S11: Quality assessment of RCTs using Cochrane RoB 2.0

Clinical trial name or acronym	Domain 1: Randomisation process	Domain 2: Deviations from intended interventions	Domain 3: Risk of bias due to missing outcome data	Domain 4: Risk of bias in measurement of the outcome	Domain 5: Risk of bias in selection of the reported result	Algorithm's overall risk of bias judgement	Assessor's overall risk of bias judgement
**ALPHA**	Low	Low	Low	Low	Low	Low	Low
**Study 301**	Some concerns	Low	Low	Low	Low	Some concerns	Low
**APPLY-PNH**	Low	Low	Low	Low	Low	Low	Low
**COMMODORE 2**	Some concerns	Low	Low	Low	Low	Some concerns	Low
**PEGASUS**	Low	Low	Low	Low	Low	Low	Low
**PRINCE**	Some concerns	Low	Low	Low	Low	Some concerns	Some concerns
**TRIUMPH**	Some concerns	Low	Low	Low	Low	Some concerns	Some concerns
**NCT04058158** Jang 2023	Low	Low	Low	Low	Low	Low	Low

Abbreviations: RCT, randomised controlled trial; RoB, risk of bias.

Supplementary Table S12: Quality assessment of single-arm trials using a modified version of the CASP checklist

Clinical trial name or acronym	Was the cohort recruited in an acceptable way?	Was the exposure accurately measured to minimise bias?	Was the outcome accurately measured to minimise bias?	Have the authors identified all important confounding factors?	Have the authors taken account of the confounding factors in the design and/or analysis?	Was the follow-up† of patients complete?	How precise (for example, in terms of confidence interval and p values) are the results?
**APPOINT-PNH**	Yes	Yes	Yes	Yes	Yes	Yes	Yes
**COMMODORE 3**	Yes	Yes	Yes	Yes	Yes	Yes	Yes
**SHEPHERD**	Yes	Yes	Yes	Unclear	Unclear	Yes	Yes

†Defined as the follow-up at the end of study duration.
Abbreviations: CASP, Critical Appraisal Skills Programme.

PRISMA checklist

Section and Topic	Item #	Checklist item	Location where item is reported
**TITLE**			
Title	1	Identify the report as a systematic review.	Page 1
**ABSTRACT**			
Abstract	2	See the PRISMA 2020 for Abstracts checklist.	Page 1
**INTRODUCTION**			
Rationale	3	Describe the rationale for the review in the context of existing knowledge.	Pages 1–2
Objectives	4	Provide an explicit statement of the objective(s) or question(s) the review addresses.	Pages 1–2
**METHODS**			
Eligibility criteria	5	Specify the inclusion and exclusion criteria for the review and how studies were grouped for the syntheses.	Table 1
Information sources	6	Specify all databases, registers, websites, organisations, reference lists and other sources searched or consulted to identify studies. Specify the date when each source was last searched or consulted.	Pages 2–3
Search strategy	7	Present the full search strategies for all databases, registers and websites, including any filters and limits used.	Supplementary Material
Selection process	8	Specify the methods used to decide whether a study met the inclusion criteria of the review, including how many reviewers screened each record and each report retrieved, whether they worked independently, and if applicable, details of automation tools used in the process.	Pages 2–3
Data collection process	9	Specify the methods used to collect data from reports, including how many reviewers collected data from each report, whether they worked independently, any processes for obtaining or confirming data from study investigators, and if applicable, details of automation tools used in the process.	Pages 2–3
Data items	10a	List and define all outcomes for which data were sought. Specify whether all results that were compatible with each outcome domain in each study were sought (e.g. for all measures, time points, analyses), and if not, the methods used to decide which results to collect.	Page 3 & Table 1
	10b	List and define all other variables for which data were sought (e.g. participant and intervention characteristics, funding sources). Describe any assumptions made about any missing or unclear information.	Table 1
Study risk of bias assessment	11	Specify the methods used to assess risk of bias in the included studies, including details of the tool(s) used, how many reviewers assessed each study and whether they worked independently, and if applicable, details of automation tools used in the process.	Pages 2–3
Effect measures	12	Specify for each outcome the effect measure(s) (e.g. risk ratio, mean difference) used in the synthesis or presentation of results.	NA
Synthesis methods	13a	Describe the processes used to decide which studies were eligible for each synthesis (e.g. tabulating the study intervention characteristics and comparing against the planned groups for each synthesis (item #5)).	Pages 2–3
	13b	Describe any methods required to prepare the data for presentation or synthesis, such as handling of missing summary statistics, or	NA

Section and Topic	Item #	Checklist item	Location where item is reported
		data conversions.	
	13c	Describe any methods used to tabulate or visually display results of individual studies and syntheses.	NA
	13d	Describe any methods used to synthesize results and provide a rationale for the choice(s). If meta-analysis was performed, describe the model(s), method(s) to identify the presence and extent of statistical heterogeneity, and software package(s) used.	NA
	13e	Describe any methods used to explore possible causes of heterogeneity among study results (e.g. subgroup analysis, meta-regression).	NA
	13f	Describe any sensitivity analyses conducted to assess robustness of the synthesized results.	NA
Reporting bias assessment	14	Describe any methods used to assess risk of bias due to missing results in a synthesis (arising from reporting biases).	NA
Certainty assessment	15	Describe any methods used to assess certainty (or confidence) in the body of evidence for an outcome.	NA
**RESULTS**			
Study selection	16a	Describe the results of the search and selection process, from the number of records identified in the search to the number of studies included in the review, ideally using a flow diagram.	Page 14, Figure 1
	16b	Cite studies that might appear to meet the inclusion criteria, but which were excluded, and explain why they were excluded.	Page 14
Study characteristics	17	Cite each included study and present its characteristics.	Supplementary Table S1
Risk of bias in studies	18	Present assessments of risk of bias for each included study.	Supplementary Table S11
Results of individual studies	19	For all outcomes, present, for each study: (a) summary statistics for each group (where appropriate) and (b) an effect estimate and its precision (e.g. confidence/credible interval), ideally using structured tables or plots.	Pages 15–25
Results of syntheses	20a	For each synthesis, briefly summarise the characteristics and risk of bias among contributing studies.	NA
	20b	Present results of all statistical syntheses conducted. If meta-analysis was done, present for each the summary estimate and its precision (e.g. confidence/credible interval) and measures of statistical heterogeneity. If comparing groups, describe the direction of the effect.	NA
	20c	Present results of all investigations of possible causes of heterogeneity among study results.	NA
	20d	Present results of all sensitivity analyses conducted to assess the robustness of the synthesized results.	NA
Reporting biases	21	Present assessments of risk of bias due to missing results (arising from reporting biases) for each synthesis assessed.	NA
Certainty of evidence	22	Present assessments of certainty (or confidence) in the body of evidence for each outcome assessed.	NA
**DISCUSSION**			

Section and Topic	Item #	Checklist item	Location where item is reported
Discussion	23a	Provide a general interpretation of the results in the context of other evidence.	Pages 25–27
	23b	Discuss any limitations of the evidence included in the review.	Pages 25–27
	23c	Discuss any limitations of the review processes used.	Pages 25–27
	23d	Discuss implications of the results for practice, policy, and future research.	Pages 25–27
**OTHER INFORMATION**			
Registration and protocol	24a	Provide registration information for the review, including register name and registration number, or state that the review was not registered.	Page 28
	24b	Indicate where the review protocol can be accessed, or state that a protocol was not prepared.	Page 28
	24c	Describe and explain any amendments to information provided at registration or in the protocol.	NA
Support	25	Describe sources of financial or non-financial support for the review, and the role of the funders or sponsors in the review.	Page 28
Competing interests	26	Declare any competing interests of review authors.	Page 28
Availability of data, code and other materials	27	Report which of the following are publicly available and where they can be found: template data collection forms; data extracted from included studies; data used for all analyses; analytic code; any other materials used in the review.	Page 28
